# Effectiveness of national multicentric school-based health lifestyles intervention among chinese children and adolescents on knowledge, belief, and practice toward obesity at individual, family and schools' levels

**DOI:** 10.3389/fped.2022.917376

**Published:** 2022-08-18

**Authors:** Xinxin Wang, Jieyu Liu, Di Gao, Yanhui Li, Qi Ma, Li Chen, Manman Chen, Tao Ma, Ying Ma, Yi Zhang, Jianjun Yang, Yanhui Dong, Yi Song, Jun Ma

**Affiliations:** ^1^School of Public Health and Management, Ningxia Medical University, Yinchuan, China; ^2^Key Laboratory of Environmental Factors and Chronic Disease Control, Yinchuan, China; ^3^School of Public Health, Institute of Child and Adolescent Health, Peking University, Beijing, China; ^4^National Health Commission Key Laboratory of Reproductive Health, Beijing, China

**Keywords:** children and adolescents, obesity, knowledge, belief, practice, intervention

## Abstract

**Background:**

This study aims to evaluate the effectiveness of the trial of national multicentric school-based health lifestyles intervention toward childhood obesity on the KBP at individual, family and schools' levels.

**Methods:**

The national trial was a multi-centered, cluster-controlled trial, which was conducted in seven provinces from September 2013 to February 2014, aiming at preventing childhood overweight and obesity. Integrated intervention strategies focused on changing specific practice related to energy intake and expenditure, such as decreasing the consumption of sweetened fizzy drinks, increasing the consumption of vegetables, ensuring proper protein intake, reducing sedentary practice including screen time, and maintaining at least 1 h of moderate to vigorous physical activity. A total of 27,477 children and adolescents in the control group and 30,997 in the intervention group were recruited with a mean follow-up period of 6.7 months. The binomial response mixed-effects model was used for assessing the effects of the national school-based health lifestyles intervention on obesity-related KBP at students individual, parents' and schools' levels.

**Results:**

Children and adolescents in the intervention group mastered better obesity-related knowledge, and they had higher correct response rates to all questions about obesity-related knowledge compared to the control group (*P* < 0.05). In terms of obesity-related belief, individuals in the intervention group was more motivated than the control group, participants in the intervention group had higher correctness of 71.18, 52.94, and 56.60% than the control group of 68.61, 49.86, and 54.43%, (*P* < 0.05*)*. In addition, healthier habits of eating breakfast and drinking milk every day were observed in the intervention group. For the beliefs toward obesity, parents of the intervention group had higher correctness than the control group. At the same time except for the fruit consumption, other obesity-related practice in the intervention group were healthier than the control group (*P* < 0.05). Except for some beliefs and practice, the intervention effect at the parent level was not significant in other aspects.

**Conclusion:**

The obesity-related knowledge and beliefs of children and adolescents got improved significantly. However, the effects on the knowledge, beliefs and certain practices of their parents and school administrators failed to reach significance.

## Introduction

Obesity of children and adolescents was widely perceived as one of the most important public health challenges now (1). It was reported that the global age-standardized prevalence of obesity in children and adolescents increased from 0.7% in 1975 to 5.6% in 2016 in girls, and from 0.9% in 1975 to 7.8% in 2016 in boys (1). In addition, being overweight/obese contributed to the earlier onset of chronic disorders such as cardiovascular diseases, type II diabetes, and cancer (2–5), and could lead to anxiety and depression in adulthood (6). Therefore, interventions at an early stage when the feeding practice and biology of the body were relatively unchanged, was capable of preventing overweight and obesity in children (7). Recently, two large, rigorously designed cluster randomized clinical trials (RCTs) from the UK (8, 9) found that school-based interventions did not bring about reductions in BMI or other indices of obesity. Therefore, we tried to use a biopsychosocial framework that is different from previous interventions for childhood obesity intervention. The biopsychosocial framework for understanding obesity showed that the biological, psychosocial, and practical factors greatly contributed to the weight status of children, including the demographic and family characteristics, lifestyles, and environment of children (10, 11). For example, the ultra-processed, energy-dense, and nutrient-poor foods exposure (12), as well as the declined opportunities for physical activity could lead to an unhealthy lifestyle and subsequent adverse health outcomes (13). Thus, the intervention of childhood obesity needed to focus on individual intervention and establish an environment construction support assisted by family and school.

Previous multiple obesity interventions programs for children and adolescents had little or no obvious effect. Most of the studies were from Europe and the United States, and only a small amount were from low–and middle-income countries (14). Based on our previous findings, we found that the 6-month school-based obesity intervention in China yielded a small to null effect on obesity and hypertension (15). Consistent with previous evidence conducted in the UK (9) and United States (16), no effects were found for the obesity-related intervention on preventing overweight or obesity among children and adolescents. These findings could be attributed to the relatively short time, in which the health markers could not be changed essentially. In addition, some potential causes could be also considered: methodological limitations, low quality of design, research results limited to a local city, and the intervention limited to single-dimensional (17, 18).

The basic assumptions of the knowledge, belief and practice (KBP) health education model were that people's beliefs about whether or not they were susceptible to disease, and their perceptions of the benefits of trying to avoid it, influenced their readiness to act (19). The KBP has four major constructs namely: perceived susceptibility, perceived severity, perceived benefits, and perceived barriers; and three additional constructs which are modifying variables, cues to action and self-efficacy. Perceived susceptibility is an individual's appraisal of the risk of developing a health problem. The model assumes that a person who perceives the likelihood of being at risk to develop a disease will engage in behaviors to decrease risk. KBP health education model has always been an important theoretical basis for obesity intervention in children and adults' population, and the KBP health education model was proved to be effective in intervening obesity in previous studies (20). However, the KBP model could not only aim at the intervened students themselves, but needed the support of multiple subjects, such as parents in the family and school managers, so as to build a supportive environment for obesity intervention from many aspects. Schools were regarded as one effective setting for childhood obesity interventions, as children and adolescents were always engaged in formal education and activities with their peers in schools (21, 22). Such interventions were often incorporated methods such as health education, providing healthy school meals, parental involvement, and community engagement (23, 24). In addition to formal school education, family-based practical weight management interventions were the main approach to achieving weight control for children and adolescents (25). Since children often imitated and practiced the models from their parents' dietary practice, thus, encouraging practical changes in the whole family could also provide a supportive environment for family lifestyle changes (26, 27). Additionally, family-focused nutrition interventions were recognized as a promising intervention approach to manage childhood obesity (28, 29). However, few studies attempted to shift the school and family environments, key settings for childhood overweight and obesity.

We used The Health Lifestyles Intervention in Chinese Children and Adolescents (HLI-CCA), which was a multi-centered, cluster-controlled, school-based intervention with family and school participation aiming to prevent overweight and obesity in children and adolescents. It included elements of education around nutrition and physical activity as well as modifications to school environments and engagement of families (30). For this reason, we focused on the effectiveness of the comprehensive obesity intervention on obesity-related KBP changes at students, parents and schools' levels, aiming to provide a basis for reducing childhood obesity.

## Materials and methods

### Study design and participants chosen

This national trial with a multi-centered, cluster-controlled trial design took place in seven provinces (centers) including Hunan, Ningxia, Tianjin, Chongqing, Liaoning, Shanghai, and Guangdong from September 2013 to February 2014. The full trial protocol has been published previously in detail (30). A multi-stage whole-group sampling method was used to select several districts from each province/municipality randomly, 12–16 primary and secondary schools were randomly selected from each district, two classes were randomly selected at each grade level, and the whole class of students and their parents were invited to participate in this survey. According to the medical history and physical examination data, we excluded participants with diseases of heart, lung, kidney, and other important organs, abnormal physical development, physical impairment, and body deformity. In the end, 92 schools were included in our study and were assigned to the control or the intervention group (the student's number: primary school: secondary school = 1:1; intervention: controlled = 1:1; urban: rural =1:1). Students who signed the informed consent form participated in this study for physical measurements and questionnaire surveys. The same protocol was used for all survey sites during the implementation process. The project was approved by the Ethical Committee of Peking University (IRB No. 00001052-12072).

Based on the trial protocol, a planned sample size of 7,000 had a 90% ability to detect a 10% difference in the obesity intervention group with a 5% statistical significance (two-sided) (30). A sample size of 5,000 had an 85% ability to detect a 10% difference.

### Intervention

**Figure 1:**
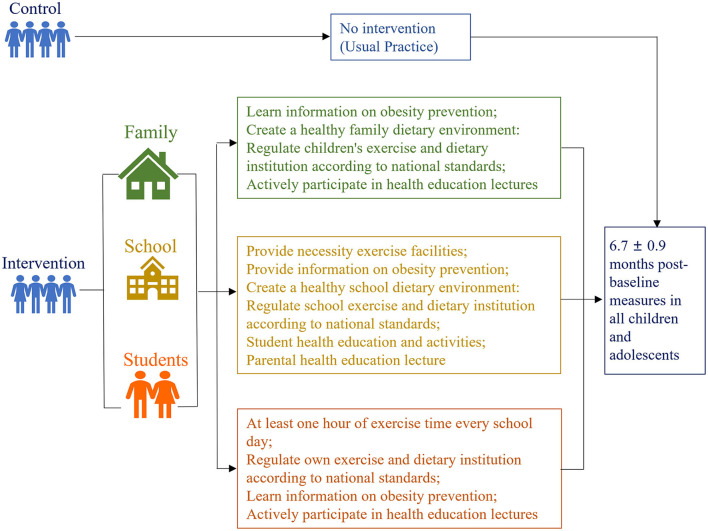
Comprehensive intervention strategy of the HLI-CCA.

**Figure 2:**
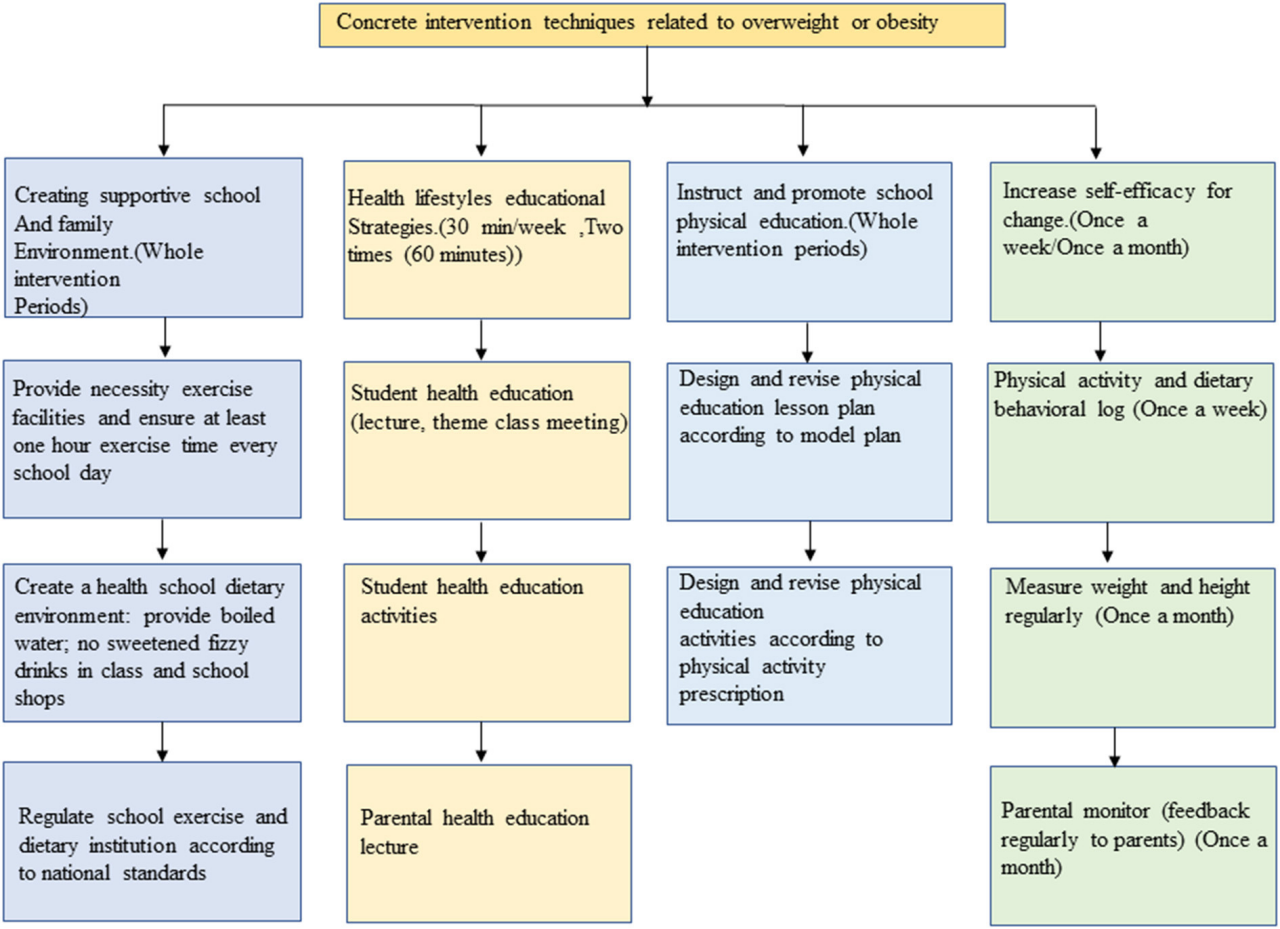
The monitoring and instruction of obesity related behaviors.

The HLI-CCA aimed to deliver a general healthy lifestyle message encouraging a healthy energy balance. The intervention consisted of four components: (a) Create supportive school and family environment, (b) Health lifestyles education and related compulsory physical activities, (c) Instruct and promote school physical education, d) Self-monitor obesity related behaviors. In schools assigned to the intervention, the HLI-CCA was delivered to children and adolescents with integrated intervention strategies focused on changing specific practice related to energy intake and expenditure. We used the “52110” method to manage children's daily behavior. ‘5', indicated the daily intake of fruits and vegetables should reach the size of 5 adults' fists. ‘2', suggested refers to watching TV or playing video games for <2 h. ‘1', indicated do more than 1 h of moderate or high-intensity physical activity every day. ‘1', meant another 1 is to eat no more than 1 serving of meat per day. The amount of 1 serving here is equivalent to the size and thickness of an adult's palm (excluding fingers), about 80–110 grams. 0, 0 indicated avoiding sugar-sweetened beverages. We carried out health education for students in the form of health education lectures and theme class meetings, including weekly health education lectures (30 min/week) and two theme class meetings (2 times 60 min). Reducing sedentary practice including screen time, physical exercise time (including physical education class, morning exercise/inter-class exercise, extracurricular activities) reached 1 h/day, and the intensity of physical exercise was above medium. Integrating the above intervention elements, the comprehensive intervention strategy of the HLI-CCA includes the following three parts during the whole intervention period (Figure 1), concrete intervention techniques related to overweight and obesity (Figure 2), which were described in the previously published protocol in detail (30).

We targeted the school and family environments through a few strategies for creating a supportive environment of both physical activity and healthier dietary habits. Elements included the provision were published elsewhere (15, 30). In the schools of control group, no specific intervention strategies or activities were carried out throughout the study period, but only records of their daily activities according to ordinary monitoring were conducted by project members. Project members, school managers, class teachers, school doctors, parents, and students themselves were all involved in the intervention project. All schools completing the trial would be provided with all intervention materials from the project team to reduce loss of follow-up. The investigators arranged to supervise the intervention in schools throughout the program. Detailed quality control measures and process evaluation were implemented according to the protocol, including the specialized intervention manual, professional training for all the intervention school doctors, health education teachers, and physical education teachers, routine supervision by project managers, and appropriate incentives to avoid loss of follow-up. For example, all schools completing the trial would be offered all the intervention materials from the project team and they would be honored as “model schools” by the Education Bureau. The class teachers and school doctors involved would receive some financial charges, and students would receive some presents.

### Data collection

Information on students' basic characteristics (e.g., age, sex), knowledge and belief, feeding practice, physical activity, etc., was obtained through student and parental questionnaires, and information on obesity-related management of school was also collected through school questionnaires. Both parental and children's questionnaires of children grade 1–3 were reported by parents. We found in the pre-experiment that parents were very aware of their children's behaviors and were able to respond correctly to their children's behaviors. Meanwhile, the questionnaire was filled out according to the children's answers. Therefore, the influence of parents filling in the questionnaire was largely reduced. Children above the fourth grade would fill in children's questionnaire by themselves instructed by the class teacher. The physical examination was carried out according to the operational specifications of the “2010 China Student Physical Fitness and Health Survey Report” (31). Information on students' height and weight was obtained. Height was measured using the portable stadiometer with 0.1 cm precision, weight was measured using a Lever type weight scale to the nearest 0.1 kg. Every indicator was measured twice, and the average level of the two measurements were calculated for final analyses. BMI was calculated as body weight (kg) divided by height (m) squared.

### Index definition

The type of obesity was determined in combination with the national health industry standard. In accordance with the guideline of screening for overweight and obesity among school-age children and adolescents (WS/T586-2018) (32), those with BMI ≥ the “overweight” or “obesity” threshold of corresponding sex and age group are defined as overweight and obesity. We also used two other criteria [International Obesity Task Force (IOTF) criteria (33) and WHO criteria (34)] for overweight and obesity in children and adolescents.

Information of obesity-related KBP was collected using standardized self-administrated questionnaire. The obesity-related knowledge and practice collection of students' questionnaires and the parental questionnaire were the same. Obesity-related knowledge included four questions on dietary habits and physical activities. The responses of all items were: “true,” “false,” or “don't know.” The answers of obesity-related beliefs questions were: “yes,” “no.” Obesity-related practice included 4 questions on dietary lifestyles. As previously published (35, 36), the frequency (days) and amount (serving per day) of dietary practice, including the total consumption of fruits and sugar-sweetened beverages (SSBs) over the past 7 days were investigated, participants were asked “How many days, over the past 7 days, have you eaten fruits or drunk SSBs? How many servings in 1 day?”. To better understand of the intake of fruits, one serving was defined as the size of an ordinary adult's closed fist, which had been described previously (37). One serving of SSB was determined as a canned beverage (~250 ml). The daily consumption of fruits and SSBs for each participants was calculated as follows: [frequency of consumption (days) × amount in each of those days (servings)] / 7. For breakfast and milk intake, participants were asked “How many days, over the past 7 days, have you eaten breakfast?” and “How many days, over the past 7 days, have you drink milk?”. Dietary consumption (fruit, breakfast, SSBs and milk) was judge as whether children's food consumption met their need according to the dietary guidelines for school-age children (38).

For parental questionnaire, there were four questions on obesity-related beliefs, all of which were answered: “yes,” “no.” In terms of school's questionnaire, there were one questions on knowledge related to obesity with the answers: “true,” “false” or “don't know” and one question on belief related to obesity with the answers: “yes,” “no.” In addition, two practice of schools related to obesity were asked, regarding arranging sports activities in the afternoon and not providing SSBs in the school canteen, with the responses: “yes” and “no.” Arranging sports activities was defined as: schools should provide necessity exercise facilities and ensure at least 1-h exercise time every school day.

### Statistical analysis

Description of baseline information was expressed as mean ± SD for continuous variables and number (%) for categorical variables. Mixed-effects regression model analysis was used to evaluate the impact of the intervention on the categorical variables, and was applied to calculate the odds ratio (OR) and 95% confidence interval (95% CI) after adjusting for age, sex, province, and urban/rural area, with values >1 indicating an increase in the effective effect of the variable following the intervention and values <1 indicating a decrease in the effective effect, with significant *P*-values.

A stratified analysis based on baseline urban and rural areas was used to explore which causes were sensitive to the intervention. Considering the age composition of our primary and secondary school levels, in our study, the subgroup analysis method was adopted, and grouping analysis was conducted according to three age groups, 6–10, 11–14, and 15–18 years old, to determine the effective or sensitive subgroups of the intervention.

All analyses were performed with SPSS 25.0 (IBM SPSS Statistics 25.0, USA) and Stata version14.0. A two-sided *P* < 0.05 was considered statistically significant.

## Results

### Project participants

Baseline information was presented in Table 1. The control and intervention groups consisted of 30,997 and 27,477 participants, respectively, of whom 51.2 and 51.2% were boys. The mean age for the control and intervention groups was 11.0 ± 3.3 and 10.7 ± 3.3 years, respectively. In addition, significant differences were found between the two groups regarding single-child status, parental education levels, parental occupations, and monthly household income (*P* < 0.05). Lower levels of height, weight, BMI, hip circumference, and waist circumference were observed for control groups, compared with the intervention group (*P* < 0.05). According to the Chinese standard, IOTF standard and WHO standard, the prevalence of overweight/obesity was 23.2/24.0%, 20.0/20.6% and 24.4/25.5% for the intervention and control groups, respectively.

**Table 1:** Baseline characteristics of study population.

**Characteristics**	**Intervention**	**Control**	***P-*value**
N	30,997	27477	
Boys (%)	51.2	51.2	0.963
Age (years)	11.0 (3.3)	10.7 (3.3)	<0.000
Urban area (%)	19,780 (63.8)	17,376 (63.2)	0.15
**Socio-demographics**, ***n*** **(%)**			
Single-child status	21,351 (68.9)	18,702 (68.1)	0.034
Parental educational level			
Paternal senior high school or above	16,188 (59.1)	13,369 (53.8)	<0.001
Maternal senior high school or above	15,133 (55.3)	12,318 (49.7)	<0.001
Paternal occupation			
Administrator and clerk	1,820 (8.2)	1277 (6.5)	<0.001
Professional and technical	3,336 (15.1)	2,790 (14.1)	
Commerce and services	7,654 (34.7)	6,596 (33.4)	
Other	9,271 (42.0)	9,066 (46.0)	
Maternal occupation			
Administrator and clerk	879 (3.8)	555 (2.6)	<0.001
Professional and technical	3,028 (12.9)	2,483 (11. 8)	
Commerce and services	8,340 (35.6)	7,313 (34.7)	
Other	11,199 (47.8)	10,728 (50. 9)	
Monthly household income (RMB)			
<2,000	2,319 (16.7)	2,360 (18.0)	0.02
2,000–5,000	6,845 (49.3)	6,404 (48.8)	
More than 5,000 or refused to answer	4,708 (33.9)	4,363 (33.2)	
**Anthropometry and prevalence**, ***n*** **(%)/mean (SD)**			
HT, mean (SD) (cm)	147.0 (17.1)	144.9 (16.9)	<0.001
WT, mean (SD) (kg)	41.7 (15.5)	40.4 (15.6)	<0.001
BMI, mean (SD) (kg/m^2^)	18.7 (3.8)	18.6 (3.8)	0.002
HC, mean (SD) (cm)	77.9 (12.1)	76.6 (12.1)	<0.001
WC, mean (SD) (cm)	65.3 (10.9)	64.6 (10.9)	<0.001
(Chinese standard) Overweight and obesity, *n* (%)	6,965 (23.2)	6,385 (24.0)	0.031
(IOTF) Overweight and obesity, n (%)	6,217 (20.0)	5,704 (20.6)	0.043
(WHO) Overweight and obesity, n (%)	7,598(24.4)	7,036(25.5)	0.004

*HT, height; WT, weight; BMI, body mass index; HC, hip circumference; WC, waist circumference*.

### Effectiveness of intervention on children and adolescents' KBP

As shown in Table 2, after a mean 6-months of the intervention, children and adolescents in the intervention group mastered better obesity-related knowledge, with the correctness of 92.17, 82.24, and 44.21% in the intervention group compared to 90.89, 81.30, and 40.18% in the control group. In the question of “It is necessary to take exercise every day,” “The food at the bottom of the dietary pagoda should be eaten more,” and “Obesity is bad for health,” the correctness increased after the intervention, and the differences were statistically significant (all *P* < 0.05). Concerning obesity-related beliefs, participants in the intervention group had higher correctness of 71.18, 52.94, and 56.60% than the control group of 68.61, 49.86, and 54.43%, respectively, with all *P* levels <0.05. In terms of feeding practice, the practice of having breakfast (intervention: 85.69%; control: 83.74%) and drinking milk every day (intervention: 46.19%; control: 42.81%) improved, with differences being statistically significant (*P* < 0.001). Children and adolescents in the rural intervention group improved obesity-related knowledge and feeding practice. It was worth noting that in the intervention group, girls were better than boys in terms of obesity-related beliefs. However, 6–10 years children and adolescents, and single-children had better knowledge about obesity, but in practice children and adolescents aged 11–15 and non-only-child children did better (Table 3; Supplementary Table 1). There was not much difference between urban and rural areas in terms of belief (Supplementary Table 2).

**Table 2:** Effectiveness on the student's knowledge, belief, and practice.

**Indicators**	**Time**	**Boy**		**Girls**		**Total**	
		**Intervention**	**Control**	**Intervention**	**Control**	**Intervention**	**Control**
**Knowledge**							
It is necessary to exercise every day [yes]	Baseline (%)	90.32	89.29	90.27	90.19	89.90	89.73
	Post-intervention (%)	91.10	89.82	93.27	91.98	92.17	90.89
	Change (%)	0.78	0.53	3.00	1.79	2.27	1.16
	OR (95% CI)	**1.15 (1.02–1.31)**		**1.23 (1.08–1.42)**		**1.19 (1.08–1.30)**	
	*p*-values	**0.025**		**0.003**		**<0.001**	
It is healthier to drink plain boiled water than	Baseline (%)	82.91	83.88	86.43	86.09	84.64	84.96
sugar-sweetened beverages [yes]	Post-intervention (%)	81.21	80.43	83.30	82.19	82.24	81.30
	Change (%)	−1.70	−3.45	−3.13	−3.90	−2.40	−3.66
	OR (95% CI)	**1.14 (1.04–1.26)**		1.06(0.95**–**1.17)		**1.10 (1.03–1.18)**	
	*p*-values	**0.006**		0.287		**0.006**	
The food at the bottom of the dietary pagoda	Baseline (%)	34.23	32.96	33.42	32.62	33.83	32.79
should be eaten more [yes]	Post-intervention (%)	45.27	41.00	43.13	39.33	44.21	40.18
	Change (%)	11.04	8.04	9.71	6.71	10.38	7.39
	OR (95% CI)	**1.19 (1.09–1.30)**		**1.20 (1.10–1.32)**		**1.20 (1.12–1.27)**	
	*p*-values	**<0.001**		**<0.001**		**<0.001**	
Obesity is bad for health [yes]	Baseline (%)	79.73	76.59	81.46	79.19	80.59	77.88
	Post-intervention (%)	83.57	77.03	86.02	79.43	84.79	78.22
	Change (%)	3.84	0.44	4.56	0.24	4.20	0.34
	OR (95% CI)	**1.36 (1.23–1.51)**		**1.56 (1.40–1.74)**		**1.45 (1.35–1.56)**	
	*p*-values	**<0.001**		**<0.001**		**<0.001**	
**Belief**							
Believe you can achieve an ideal weight	Baseline (%)	67.77	68.09	67.16	66.76	67.47	67.44
status through effort [true]	Post-intervention (%)	71.24	69.40	71.11	67.79	71.18	68.61
	Change (%)	3.47	1.31	3.95	1.03	3.71	1.17
	OR (95% CI)	**1.14 (1.04–1.24)**		**1.20 (1.10–1.30)**		**1.17 (1.10–1.24)**	
	*p*-values	**0.003**		**<0.001**		**<0.001**	
Eat more fruits and vegetables and less	Baseline (%)	42.92	42.01	50.11	48.30	46.48	45.13
high-energy snacks to lose weight [true]	Post-intervention (%)	49.15	46.88	56.81	52.89	52.94	49.86
	Change (%)	6.23	4.87	6.70	4.59	6.46	4.73
	OR (95% CI)	1.08 (0.99**–**1.18)		**1.13 (1.04–1.23)**		**1.11 (1.04–1.18)**	
	*p*-values	0.068		**0.005**		**0.001**	
Exercise to lose weight [true]	Baseline (%)	50.44	50.43	48.22	48.06	49.34	49.26
	Post-intervention (%)	55.92	54.79	57.28	54.07	56.60	54.43
	Change (%)	5.48	4.36	9.06	6.01	7.26	5.17
	OR (95% CI)	1.07 (0.98**–**1.16)		**1.19 (1.09–1.30)**		**1.13 (1.06–1.20)**	
	*p*-values	0.128		**<0.001**		**<0.001**	
**Practice**							
Fruit [daily intake≥3 servings/day].	Baseline (%)	8.51	7.90	8.50	7.39	8.50	7.65
	Post-intervention (%)	10.37	8.61	9.72	7.75	10.05	8.19
	Change (%)	1.86	0.71	1.22	0.36	1.55	0.54
	OR (95% CI)	**1.17 (1.02–1.34)**		1.12 (0.98**–**1.29)		**1.14 (1.04–1.26)**	
	*p*-values	**0.025**		0.107		**0.007**	
Sugar-sweetened beverages [daily intake = 0	Baseline (%)	29.55	29.90	36.17	35.78	32.81	32.80
cups/week]	Post-intervention (%)	33.60	32.56	40.66	40.29	37.09	36.37
	Change (%)	4.05	2.66	4.49	4.51	4.28	3.57
	OR (95% CI)	1.09(1.00**–**1.19)		1.00(0.92**–**1.09)		1.04(0.98**–**1.11)	
	*p*-values	0.050		0.983		0.175	
Breakfast [frequency = 7 day/week]	Baseline (%)	84.33	83.83	83.92	82.12	84.13	83.48
	Post-intervention (%)	85.33	84.07	86.07	83.40	85.69	83.74
	Change (%)	1.00	0.24	2.15	1.28	1.56	0.26
	OR (95% CI)	1.09 (0.97**–**1.23)		**1.26 (1.12–1.42)**		**1.17 (1.08–1.27)**	
	*p*-values	0.133		**<0.001**		**<0.001**	
Milk [frequency = 7 day/week]	Baseline (%)	43.92	44.72	41.18	41.33	42.57	43.05
	Post-intervention (%)	49.97	44.49	44.35	41.07	46.19	42.81
	Change (%)	6.05	−0.23	3.17	−0.26	3.62	−0.24
	OR (95% CI)	**1.30 (1.19–1.42)**		**1.24 (1.14–1.36)**		**1.27 (1.20–1.35)**	
	*p*-values	**<0.001**		**<0.001**		**<0.001**	

*Model was adjusted for age, sex, province and urban/rural area. Bold values indicated the statistically significant values for the effect for OR with p <0.05*.

**Table 3:** Effectiveness on the student's knowledge, belief, and practice (by age).

**Indicators**	**Time**	**6–10 (years)**		**11–14 (years)**		**15–18 (years)**		**Total**	
		**Intervention**	**Control**	**Intervention**	**Control**	**Intervention**	**Control**	**Intervention**	**Control**
**Knowledge**									
It is necessary to exercise every day [yes]	Baseline (%)	89.29	89.30	91.30	90.98	89.39	89.43	89.90	89.73
	Post-intervention (%)	92.20	89.81	92.97	92.84	91.08	90.92	92.17	90.89
	Change (%)	2.91	0.51	1.67	1.86	1.69	1.49	2.27	1.16
	OR (95% CI)	**1.40 (1.24–1.59)**		0.96 (0.79–1.16)		1.02 (0.82–1.26)		**1.19 (1.08–1.30)**	
	*p*-values	**<0.001**		0.668		0.876		**<0.001**	
It is healthier to drink plain boiled water than sugar-sweetened	Baseline (%)	83.03	83.55	84.59	85.06	88.78	89.02	84.64	84.96
beverages. [yes]	Post-intervention (%)	81.27	79.62	81.67	81.88	85.09	84.56	82.24	81.30
	Change (%)	−1.76	−3.93	−2.92	−3.18	−3.69	−4.46	−2.40	−3.66
	OR (95% CI)	**1.17 (1.07–1.29)**		1.02 (0.89–1.16)		1.08 (0.90–1.29)		**1.10 (1.03–1.18)**	
	*p*-values	**0.001**		0.775		0.409		**0.006**	
The food at the bottom of the dietary pagoda should be eaten	Baseline (%)	32.33	31.67	34.81	33.53	36.20	35.27	33.83	32.79
more [yes]	Post-intervention (%)	41.15	37.62	49.78	43.89	42.18	42.13	44.21	40.18
	Change (%)	8.82	5.95	14.97	10.36	5.98	6.86	10.38	7.39
	OR (95% CI)	**1.20 (1.10–1.31)**		**1.27 (1.12–1.43)**		0.96 (0.83–1.12)		**1.20 (1.12–1.28)**	
	*p*-values	**<0.001**		**<0.001**		0.634		**<0.001**	
Obesity is bad for health [yes]	Baseline (%)	83.84	81.69	77.83	73.85	76.43	72.24	80.59	77.88
	Post-intervention (%)	88.55	81.27	83.82	76.96	78.69	73.10	84.79	78.22
	Change (%)	4.71	−0.42	5.99	3.11	2.26	0.86	4.20	0.34
	OR (95% CI)	**1.70 (1.52–1.89)**		1.38 (1.19–1.57)		1.14 (0.97–1.34)		**1.45 (1.35–1.56)**	
	*p*-values	**<0.001**		**<0.001**		0.118		**<0.001**	
**Belief**									
Believe you can achieve an ideal weight status through effort [true]	Baseline (%)	69.02	69.60	66.72	66.82	64.70	62.00	67.47	67.44
	Post-intervention (%)	71.40	70.03	71.66	69.32	68.14	63.85	71.18	68.61
	Change (%)	2.38	0.43	4.94	2.50	3.44	1.85	3.71	1.17
	OR (95% CI)	**1.19 (1.09–1.29)**		**1.18 (1.06–1.34)**		1.10 (0.95–1.26)		**1.17 (1.10–1.24)**	
	*p*-values	**<0.001**		**0.004**		0.194		**<0.001**	
Eat more fruits and vegetables and less high-energy snacks to	Baseline (%)	44.17	42.72	48.66	50.71	49.02	44.66	46.48	45.13
lose weight [true]	Post-intervention (%)	50.14	47.31	56.33	52.38	53.35	51.71	52.94	49.86
	Change (%)	5.97	4.59	7.67	1.67	4.33	7.05	6.46	4.73
	OR (95% CI)	1.07 (0.99–1.17)		**1.46 (1.29–1.64)**		0.84 (0.72–0.975)		**1.11 (1.04–1.18)**	
	*p*-values	0.092		**<0.001**		0.015		**0.001**	
Exercise to lose weight [true]	Baseline (%)	48.82	48.84	53.00	54.66	45.31	42.95	49.34	49.26
	Post-intervention (%)	55.73	53.61	61.10	58.92	51.25	49.69	56.60	54.43
	Change (%)	6.91	4.77	8.10	48.23	5.94	6.74	7.26	5.17
	OR (95% CI)	**1.12 (1.03–1.22)**		**1.26 (1.12–1.42)**		0.95 (0.83–1.10)		**1.13 (1.06–1.20)**	
	*p*-values	**0.006**		**<0.001**		0.509		**<0.001**	
**Practice**	Baseline (%)	8.82	7.52	9.54	9.90	6.22	4.91	8.50	7.65
Fruit [daily intake≥3 servings/day]	Post-intervention (%)	10.73	8.25	11.69	10.05	6.16	5.28	10.05	8.19
	Change (%)	1.91	0.73	2.15	0.15	−0.06	0.37	1.55	0.54
	OR (95% CI)	**1.15 (1.01–1.31)**		**1.29 (1.08–1.53)**		0.90 (0.69–1.19)		**1.14 (1.04–1.26)**	
	*p*-values	**0.041**		**0.004**		0.479		**0.007**	
	Baseline (%)	36.76	36.92	30.94	29.91	25.71	24.57	32.81	32.80
Sugar-sweetened beverages [daily intake = 0 cups/week]	Post-intervention (%)	40.01	41.09	35.71	31.55	33.42	32.66	37.09	36.37
	Change (%)	3.25	4.17	4.77	1.64	7.71	8.09	4.28	3.57
	OR (95% CI)	0.96 (0.88–1.04)		**1.25 (1.11–1.41)**		0.94 (0.81–1.10)		1.04 (0.98–1.11)	
	*p*-values	0.293		**<0.001**		0.445		0.171	
Breakfast [frequency = 7 days/week]	Baseline (%)	91.31	91.15	77.81	74.48	75.30	73.02	84.13	83.48
	Post-intervention (%)	92.44	91.79	81.96	76.21	77.81	75.56	85.69	83.74
	Change (%)	1.13	0.64	4.15	1.73	2.51	2.54	1.56	0.26
	OR (95% CI)	1.10 (0.95–1.27)		**1.35 (1.17–1.55)**		1.01 (0.86–1.19)		**1.17 (1.08–1.27)**	
	*p*-values	0.191		**<0.001**		0.870		**<0.001**	
Milk [frequency = 7 days/week]	Baseline (%)	52.93	48.06	42.06	41.45	32.06	30.27	42.57	43.05
	Post-intervention (%)	48.93	46.10	48.61	44.72	37.26	32.83	46.19	42.81
	Change (%)	−4.00	−1.96	6.55	3.27	5.20	2.56	3.62	−0.24
	OR (95% CI)	**1.26 (1.16–1.37)**		**1.24 (1.10–1.39)**		**1.20 (1.03–1.40)**		**1.27 (1.20–1.35)**	
	*p*-values	**<0.001**		**0.001**		**0.017**		**<0.001**	

*Model was adjusted for sex, province and urban/rural area. Bold values indicated the statistically significant values for the effect for OR with p <0.05*.

### Effectiveness of intervention on the parents' KBP

There was no significant difference between the intervention and control groups in terms of knowledge about the obesity toward children among parents (Tables 4, 5).

**Table 4:** Effectiveness of intervention on the parents' knowledge, belief, and practice.

**Indicators**	**Time**	**Boy**		**Girls**		**Total**	
		**Intervention**	**Control**	**Intervention**	**Control**	**Intervention**	**Control**
**Knowledge**							
It is necessary to exercise every day [yes]	Baseline (%)	89.83	88.59	90.41	90.05	90.12	89.33
	Post-intervention (%)	89.86	89.61	91.68	91.26	90.77	90.43
	Change (%)	0.03	1.02	1.27	1.21	0.65	1.10
	OR (95% CI)	0.89 (0.78–1.01)		1.02 (0.89–1.17)		0.95 (0.86–1.04)	
	*p*-values	0.064		0.811		0.243	
It is healthier to drink plain boiled water than	Baseline (%)	94.74	94.55	94.52	94.28	94.63	94.42
sugar-sweetened beverages [yes]	Post-intervention (%)	92.58	91.90	91.53	91.68	92.05	91.79
	Change (%)	−2.16	−2.65	−2.99	−2.60	−2.58	−2.63
	OR (95% CI)	1.06 (0.91–1.24)		0.94 (0.81–1.09)		1.00 (0.89–1.11)	
	*p*-values	0.436		0.387		0.930	
The food at the bottom of the dietary pagoda	Baseline (%)	33.26	32.69	35.18	32.64	34.22	32.66
should be eaten more [yes]	Post-intervention (%)	40.74	39.14	40.01	39.14	40.37	39.14
	Change (%)	7.48	6.45	4.83	6.50	6.15	6.48
	OR (95% CI)	1.06 (0.97–1.16)		0.90 (0.82–0.98)		0.98 (0.91–1.04)	
	*p*-values	0.201		0.019		0.44	
Obesity is bad for health [yes]	Baseline (%)	87.77	86.78	88.04	86.76	87.91	86.77
	Post-intervention (%)	87.20	86.53	88.65	87.05	87.93	86.79
	Change (%)	−0.57	−0.25	0.61	0.29	0.02	0.02
	OR (95% CI)	0.96 (0.85–1.09)		1.04 (0.92–1.18)		1.00 (0.92–1.09)	
	*p*-values	0.535		0.501		0.985	
**Belief**							
Don't buy drinks for children [true]	Baseline (%)	43.72	44.62	44.36	45.86	44.04	45.24
	Post-intervention (%)	47.38	45.77	47.77	47.68	47.57	46.72
	Change (%)	3.66	1.15	3.41	1.82	3.53	1.48
	OR (95% CI)	**1.15 (1.05–1.25)**		**1.09 (1.00–1.19)**		**0.98 (1.06–1.19)**	
	*p*-values	**0.001**		**0.040**		**<0.001**	
Children take part in physical exercise with their	Baseline (%)	77.36	79.15	72.93	74.89	75.15	77.03
classmates [true]	Post-intervention (%)	81.64	80.43	76.25	77.02	78.95	78.73
	Change (%)	4.28	1.28	3.32	2.13	3.80	1.70
	OR (95% CI)	**1.26 (1.14–1.40)**		1.08 (0.98–1.19)		**1.16 (1.08–1.24)**	
	*p*-values	**<0.001**		0.118		**<0.001**	
Set aside time every day for children to	Baseline (%)	82.42	83.31	78.31	81.30	80.36	82.31
exercise [true]	Post-intervention (%)	85.47	85.34	82.07	83.26	83.77	84.31
	Change (%)	3.05	2.03	3.76	1.96	3.41	2
	OR (95% CI)	1.09 (0.98–1.22)		**1.14 (1.03–1.27)**		**1.12 (1.03–1.21)**	
	*p*-values	0.115		**0.015**		**0.005**	
Look at the nutrition label when shopping in the		70.99	71.63	71.34	72.22	71.17	71.93
supermarket [true]	Baseline (%)	74.65	73.52	75.05	74.90	74.85	74.20
	Post-intervention (%)	3.66	1.89	3.71	2.68	3.68	2.27
	Change (%)	**1.13 (1.03–1.24)**		1.07 (0.97–1.18)		**1.01 (1.03–1.18)**	
	OR (95% CI)	**0.012**		0.161		**0.006**	
Drinks are not always available at home [true]	*p*-values	67.65	67.95	69.17	70.50	68.41	69.22
	Baseline (%)	69.31	68.50	70.43	71.27	69.87	69.88
	Post-intervention (%)	1.66	0.55	1.26	0.77	1.46	0.66
	Change (%)	0.93 (0.85–1.02)		s0.97 (0.88–1.06)		0.95 (0.89–1.01)	
	OR (95% CI)	0.129		0.500		0.118	
**Practice**	*p*-values						
Fruit [daily intake≥3 servings/day]	Baseline (%)	6.20	5.55	6.48	5.61	6.34	5.58
	Post-intervention (%)	6.72	6.75	7.19	6.45	6.96	6.60
	Change (%)	0.52	1.20	0.71	0.84	0.62	1.02
	OR (95% CI)	0.87 (0.74–1.03)		0.96 (0.82–1.13)		0.92 (0.82–1.03)	
	*p*-values	0.097		0.630		0.130	
Sugar-sweetened beverages [daily intake ≤ 4	Baseline (%)	91.27	92.01	92.54	93.28	91.91	92.65
cups/week]	Post-intervention (%)	91.80	92.03	93.28	93.02	92.54	92.53
	Change (%)	0.53	0.02	0.74	−0.26	0.63	−0.12
	OR (95% CI)	1.08 (0.93–1.25)		**1.19 (1.02–1.39)**		**1.13 (1.02–1.26)**	
	*p*-values	0.312		**0.028**		**0.025**	
Breakfast [frequency = 7 day/week]	Baseline (%)	84.74	85.99	85.20	85.93	84.97	85.96
	Post-intervention (%)	87.43	86.57	87.70	86.70	87.57	86.64
	Change (%)	2.69	0.58	2.50	0.77	2.60	0.68
	OR (95% CI)	**1.25 (1.11–1.41)**		**1.21 (1.07–1.36)**		**1.23 (1.13–1.34)**	
	*p*-values	**<0.001**		**0.002**		**<0.001**	
Milk [frequency = 7 day/week]	Baseline (%)	28.78	29.03	27.43	26.22	28.10	27.63
	Post-intervention (%)	32.39	30.45	31.03	28.85	31.71	29.66
	Change (%)	3.61	1.42	3.60	2.63	3.61	2.03
	OR (95% CI)	**1.15 (1.05–0.96)**		1.06 (0.96–1.16)		**1.10 (1.03–1.18)**	
	*p*-values	**0.004**		0.229		**0.003**	

*Model was adjusted for age, sex, province and urban/rural area. Bold values indicated the statistically significant values for the effect for OR with p <0.05*.

**Table 5:** Effectiveness on the parents' knowledge, belief, and practice (by age).

**Indicators**	**Time**	**6–10 (years)**		**11–14 (years)**		**15–18 (years)**		**Total**	
		**Intervention**	**Control**	**Intervention**	**Control**	**Intervention**	**Control**	**Intervention**	**Control**
**Knowledge**									
It is necessary to exercise every day [yes]	Baseline (%)	89.28	89.11	91.15	89.32	90.94	90.18	90.12	89.33
	Post-intervention (%)	90.45	89.71	91.36	91.46	90.65	90.89	90.77	90.43
	Change (%)	1.17	0.60	0.21	2.14	−0.29	0.71	0.65	1.10
	OR (95% CI)	1.09 (0.96–1.23)		**0.77 (0.64–0.92)**		0.87 (0.69–1.10)		0.95 (0.86–1.04)	
	*p*-values	0.207		**0.004**		0.235		0.242	
It is healthier to drink plain boiled water than sugar-sweetened	Baseline (%)	95.34	95.00	94.92	93.89	92.09	92.68	94.63	94.42
beverages [yes]	Post-intervention (%)	94.16	93.97	93.02	90.63	85.77	85.79	92.05	91.79
	Change (%)	−1.18	−1.03	−1.90	−3.26	−6.32	−6.89	−2.58	−2.63
	OR (95% CI)	0.96 (0.81–1.12)		1.15 (0.94–1.40)		1.09 (0.86–1.37)		1.00 (0.89–1.11)	
	*p*-values	0.592		0.166		0.473		0.924	
The food at the bottom of the dietary pagoda should be eaten	Baseline (%)	33.64	32.67	34.00	29.94	36.24	38.16	34.22	32.66
more [yes]	Post-intervention (%)	38.95	39.21	41.80	37.91	41.48	42.14	40.37	39.14
	Change (%)	5.31	6.54	7.80	7.97	5.24	3.98	6.15	6.48
	OR (95% CI)	0.94 (0.86–1.03)		0.96 (0.85–1.08)		1.08 (0.92–1.27)		0.98 (0.91–1.04)	
	*p*-values	0.161		0.489		0.362		0.443	
Obesity is bad for health [yes]	Baseline (%)	90.27	88.92	87.06	84.41	82.38	80.75	87.91	86.77
	Post-intervention (%)	90.79	90.47	87.50	84.00	82.36	78.38	87.93	86.79
	Change (%)	0.52	1.55	0.44	−0.41	−0.02	−2.37	0.02	0.02
	OR (95% CI)	0.88 (0.76–1.00)		1.10 (0.94–1.28)		1.21 (0.99–1.48)		1.00 (0.92–1.09)	
	*p*-values	0.057		0.229		0.061		0.996	
**Belief**									
Don't buy drinks for children [true]	Baseline (%)	47.52	47.36	42.51	45.34	36.29	34.63	44.04	45.24
	Post-intervention (%)	52.30	50.48	46.93	45.33	37.85	35.27	47.57	46.72
	Change (%)	4.78	3.12	4.42	−0.01	1.56	0.64	3.53	1.48
	OR (95% CI)	**1.10 (1.01–1.20)**		**1.28 (1.15–1.43)**		1.05 (0.90–1.24)		**1.12 (1.05–1.19)**	
	*p*-values	**0.025**		**<0.001**		0.524		**<0.001**	
Children take part in physical exercise with their classmates [true]	Baseline (%)	77.02	79.06	74.45	75.88	71.01	69.34	75.15	77.03
	Post-intervention (%)	80.37	81.45	79.11	77.06	75.03	71.97	78.95	78.73
	Change (%)	3.35	2.39	4.66	1.18	4.02	2.63	3.80	1.70
	OR (95% CI)	1.07 (0.97–1.18)		**1.28 (1.13–1.45)**		1.11 (0.94–1.32)		**1.16 (1.08–1.24)**	
	*p*-values	0.190		**<0.001**		0.217		**<0.001**	
Set aside time every day for children to exercise [true]	Baseline (%)	81.17	83.23	79.67	81.23	79.11	79.97	80.36	82.31
	Post-intervention (%)	85.15	84.78	82.67	84.16	81.97	82.80	83.77	84.31
	Change (%)	3.98	1.55	3.00	2.93	2.86	2.83	3.41	2.00
	OR (95% CI)	**1.23 (1.11–1.37)**		1.00 (0.87–1.14)		1.00 (0.82–1.21)		**1.12 (1.03–1.21)**	
	*p*-values	**<0.001**		0.951		0.964		**0.005**	
Look at the nutrition label when shopping in the supermarket	Baseline (%)	70.93	72.33	72.51	73.22	69.64	67.30	71.17	71.93
[true]	Post-intervention (%)	74.80	74.01	76.29	75.49	72.37	71.97	74.85	74.20
	Change (%)	3.87	1.68	3.78	2.27	2.73	4.67	3.68	2.27
	OR (95% CI)	**1.16 (1.05–1.27)**		1.10 (0.97–1.24)		0.89 (0.75–1.05)		**1.09 (1.02–1.16)**	
	*p*-values	**0.003**		0.133		0.175		**0.015**	
Drinks are not always available at home [true]	Baseline (%)	69.26	69.38	68.41	70.42	65.97	65.96	68.41	69.22
	Post-intervention (%)	71.79	71.19	70.02	69.51	65.48	65.56	69.87	69.88
	Change (%)	2.53	1.81	1.61	−0.91	−0.49	−0.40	1.46	0.66
	OR (95% CI)	1.05 (0.96–1.16)		**1.19 (1.05–1.34)**		0.99 (0.84–1.17)		1.05 (0.99–1.13)	
	*p*-values	0.282		**0.005**		0.927		0.120	
**Practice**									
Fruit [daily intake≥3 servings/day]	Baseline (%)	5.58	4.93	6.92	7.13	7.67	5.74	6.34	5.58
	Post-intervention (%)	5.48	5.55	8.03	8.67	8.58	6.25	6.96	6.60
	Change (%)	−0.10	0.62	1.11	1.11	1.54	0.91	0.62	1.02
	OR (95% CI)	0.85 (0.72–1.00)		0.95 (0.78–1.15)		1.04 (0.78–1.39)		0.92 (0.82–1.03)	
	*p*-values	0.053		0.578		0.794		0.131	
Sugar-sweetened beverages [daily intake ≤ 4 cups/week]	Baseline (%)	92.62	93.11	91.06	91.98	91.24	91.71	91.91	92.56
	Post-intervention (%)	93.52	93.44	92.45	91.27	90.54	91.64	92.54	92.53
	Change (%)	0.90	0.33	1.39	−0.71	−0.70	−0.07	0.63	−0.12
	OR (95% CI)	1.11 (0.95–1.29)		**1.39 (1.15–1.67)**		0.91 (0.70–1.18)		**1.13 (1.02–1.26)**	
	*p*-values	0.188		**0.001**		0.465		**0.026**	
Breakfast [frequency = 7 days/week]	Baseline (%)	85.98	86.86	82.61	83.89	85.74	85.59	84.97	85.96
	Post-intervention (%)	88.42	87.91	87.16	84.90	86.30	85.47	87.57	86.64
	Change (%)	2.44	1.05	4.55	1.01	0.56	−0.12	2.60	0.68
	OR (95% CI)	**1.18 (1.05–1.33)**		1.07 (0.86–1.33)		**1.24 (1.04–1.48)**		**1.23 (1.13–1.34)**	
	*p*-values	**0.007**		0.558		**0.014**		**<0.001**	
Milk [frequency = 7 days/week]	Baseline (%)	28.80	28.51	25.99	25.28	29.37	27.87	28.10	27.63
	Post-intervention (%)	31.17	29.85	31.93	29.45	32.68	29.49	31.71	29.66
	Change (%)	2.37	1.34	5.94	4.17	3.31	1.62	3.61	2.03
	OR (95% CI)	1.07 (0.98–1.17)		1.12 (0.99–1.26)		1.11 (0.93–1.33)		**1.10 (1.03–1.18)**	
	*p*-values	0.155		0.074		0.244		**0.003**	

*Model was adjusted for sex, province and urban/rural area. Bold values indicated the statistically significant values for the effect for OR with p <0.05*.

For the beliefs toward obesity, parents of the intervention group had higher correctness than the control group, such as the questions of “Don't buy drinks for children,” “Children take part in physical exercise with their classmates,” “Set aside time every day for children to exercise” and “Look at the nutrition label when shopping in the supermarket” (*P* < 0.05). There was no difference in most practice items between these two groups. However, regarding some items (e.g., drinking less SSBs, eating breakfast, and drinking milk every day), the intervention group had a higher correct responses rates than the control group (*P* < 0.05) (Table 4). It was worth noting that the corrected rate of beliefs about obesity among the parents of the students in the younger age group was higher than that of the parents of the older age group (Table 5).

We found that the intervention effect on obesity-related beliefs was more pronounced for mothers working in administrator, clerk, and professional occupations (Supplementary Table 3). At the same time, the intervention effect on obesity-related behaviors improved for parents whose monthly household income was <2,000 (Supplementary Table 4). According to Table 5 and Supplementary Table 5, it could be seen that the changes in knowledge of parents in different regions and different age groups were not very different.

### Effectiveness of intervention on the obesity related KBP at school's levels

For school, the intervention on overweight and obesity had less impact on knowledge and beliefs, but the school's administrator's or measures improved, including arranging sports activities for students in the afternoon and not providing SSBs in the school canteen (Table 6). Notably, the practice of arranging sports activities for students in the afternoon in the intervention group improved better than the control group (intervention group: 6.98%; control group: 4.87%; *P* < 0.001). In contrast, the practice of not providing SSBs in the school canteen did not get satisfactory improvement after intervention in the intervention group, but was still better than the control group (*P* < 0.001). But we also found that the practice of not providing SSBs in school cafeterias in the 6–10-year-old age group did not improve after the intervention, but the intervention had a certain effect in the 11–14 and 12–18-year-old age groups (*P* < 0.001) (Table 7). It was worth noting that the school's practice change intervention on obesity has a significant effect (Supplementary Tables 6–10).

**Table 6:** Effectiveness on the school's knowledge, belief, and practice.

**Indicators**	**Time**	**Boy**		**Girls**		**Total**	
		**Intervention**	**Control**	**Intervention**	**Control**	**Intervention**	**Control**
**Knowledge**							
Teaching activities crowd out break exercises or	Baseline (%)	70.38	71.73	75.62	76.22	72.97	73.95
extracurricular sports activities [yes]	Post-intervention (%)	64.65	65.09	70.18	71.68	67.38	68.34
	Change (%)	−5.73	−6.64	−5.44	−4.54	−5.59	−5.61
	OR (95% CI)	1.06 (0.97–1.16)		0.95 (0.87–1.04)		1.01 (0.95–1.07)	
	*p*-values	0.177		0.268		0.832	
**Belief**							
The school arranges sports activities in the	Baseline (%)	8.82	8.26	7.51	7.64	8.20	7.96
afternoon [frequency≥30 min day/week [true]	Post-intervention (%)	10.52	10.31	9.78	9.73	10.16	10.03
	Change (%)	1.70	2.05	2.27	2.09	1.96	2.07
	OR (95% CI)	0.95 (0.79–1.13)		1.02 (0.84–1.23)		0.97 (0.86–1.11)	
	*p*-values	0.551		0.854		0.701	
**Practice**							
The school arranges sports activities in the	Baseline (%)	62.64	64.11	59.80	63.93	61.23	64.02
afternoon [yes]	Post-interventio*n* (%)	68.25	68.55	68.18	69.24	68.21	68.89
	Change (%)	5.61	4.44	8.38	5.31	6.98	4.87
	OR (95% CI)	1.06 (0.98–1.16)		**1.18 (1.08–1.28)**		**1.12 (1.05–1.19)**	
	*p*-values	0.149		**<0.001**		**<0.001**	
Sugar-sweetened beverages are not available in the	Baseline (%)	48.85	52.47	46.23	50.4	47.56	51.45
school canteen [yes]	Post-intervention (%)	47.66	48.04	44.86	46.29	46.28	47.17
	Change (%)	−1.19	−4.43	−1.37	−4.11	−1.28	−4.28
	OR (95% CI)	**1.35 (1.21–1.50)**		**1.32 (1.17–1.48)**		**1.33 (1.23–1.44)**	
	*p*-values	**<0.001**		**<0.001**		**<0.001**	

*Model was adjusted for age, sex, province and urban/rural area. Bold values indicated the statistically significant values for the effect for OR with p <0.05*.

**Table 7:** Effectiveness on the school's knowledge, belief, and practice (by age).

**Indicators**	**Time**	**6–10 (years)**		**11–14 (years)**		**15–18 (years)**		**Total**	
		**Intervention**	**Control**	**Intervention**	**Control**	**Intervention**	**Control**	**Intervention**	**Control**
**Knowledge**									
Teaching activities crowd out break exercises or extracurricular	Baseline (%)	72.72	75.38	74.77	74.98	71.96	68.38	72.97	73.95
sports activities	Post-intervention (%)	68.76	70.58	68.48	67.25	62.99	64.71	67.38	68.34
	Change (%)	−3.96	−4.80	−6.29	−7.73	−8.97	−3.67	−5.59	−5.61
	OR (95% CI)	1.06 (0.97–1.16)		1.12 (0.99–1.26)		0.73 (0.64–0.84)		1.01 (0.95–1.07)	
	*p*-values	0.186		0.077		<0.001		0.832	
**Attitude**									
The school arranges sports activities in the afternoon	Baseline (%)	6.02	6.19	11.97	14.53	8.63	4.34	8.20	7.96
[frequency≥30 min day/week	Post-intervention (%)	7.68	7.81	14.06	15.56	8.76	6.38	10.16	10.03
	Change (%)	1.66	1.62	2.09	1.03	0.13	2.04	1.96	2.07
	OR (95% CI)	1.01 (0.84–1.22)		1.03 (0.83–1.29)		0.81(0.55–1.18)		0.97 (0.86–1.11)	
	*p*-values	0.895		0.76		0.267		0.701	
**Practice**									
The school arranges sports activities in the afternoon	Baseline (%)	69.70	70.64	57.98	61.16	45.34	48.89	61.23	64.02
	Post-intervention (%)	70.38	72.07	70.78	70.31	60.12	59.36	68.21	68.89
	Change (%)	0.68	1.43	12.80	9.15	14.78	10.47	6.98	4.87
	OR (95% CI)	0.96 (0.88–1.04)		**1.23 (1.10–1.38)**		**1.24 (1.09–1.41)**		1.12 (1.05–1.19)	
	*p*-values	0.313		**<0.001**		**0.001**		**<0.001**	
Sugar-sweetened beverages are not available in the school canteen	Baseline (%)	73.51	71.73	31.48	36.13	6.51	12.77	47.56	51.45
	Post-intervention (%)	70.35	66.89	37.64	37.33	11.08	14.76	46.28	47.17
	Change (%)	−3.16	−4.84	6.16	1.20	4.57	1.99	−1.28	−4.28
	OR (95% CI)	**1.09 (1.00–1.18)**		**1.32 (1.19–1.46)**		**1.52 (1.27–1.84)**		**1.33 (1.23–1.44)**	
	*p*-values	**0.047**		**<0.001**		**0.001**		**<0.001**	

*Model was adjusted for sex, province and urban/rural area. Bold values indicated the statistically significant values for the effect for OR with p <0.05*.

### Subgroup analysis

Stratified analyses were conducted also using mixed-effects regression model.

The results of hierarchical analysis were similar to the main results and did not change essentially when the study sample was divided according to urban or rural areas, different ages and sociodemographic variables (Supplementary Tables 1–15).

## Discussion

To our knowledge, this was the first and the largest school-based, family-involved, comprehensive intervention trial assessing the obesity-centered intervention effectiveness toward Chinese children and adolescents on KBP at individual, parents and schools' levels. We found that the significant improvements in children and adolescents' knowledge and beliefs about obesity were observed after the mean 6-month comprehensive obesity intervention trial, but obesity-related practice were not obvious. However, the effects were not significant enough for their parents and schools' administrators knowledge, beliefs and certain. It was reasonable that practice could not change significantly over a short time, and a longer length of time to observe and more intensive interventions was required (39, 40).

This study highlighted the importance of guiding the construction of multiple environments for childhood obesity intervention and to break the obesity susceptibility environment in the real world. In this study, the significant improvements in children and adolescents' knowledge and beliefs about obesity indicated that this kind of school-based comprehensive obesity intervention was rather effective to a certain extent, but a longer time should be considered to continuously improve the physical health. The intervention study on childhood obesity in 24 schools from three different geographical regions in China showed that the intervention improved obesity-related knowledge (41), was consistent with our findings. During childhood and adolescence, knowledge, beliefs, and lifestyle practices were important modifiable factors that could curb the rising prevalence of overweight and obesity and their related consequences. Scientific evidence confirmed that, suggesting that changes in KBP could significantly impact an individual's current and future health (42, 43). Notably, improved knowledge could be a fundamental factor in regulating people's practice and habits (44, 45). According to previous findings, students were generally unfamiliar with the advice on the prevention and treatment of childhood obesity and could not identify age-appropriate cut-off points for weight status (46). Enhancing health-related knowledge was, therefore a necessary element to reduce childhood obesity effectively. Also, positive beliefs helped to improve knowledge and generate appropriate practice. Since children and adolescents spent a large part of their daily lives at school and home, it was important to apply the tripartite resources between the students themselves, their families and schools, to link up knowledge, beliefs and their practice.

Children and adolescents' poor dietary practice, including skipping breakfast, low consumption of fruits and vegetables, unhealthy snacking practice and decreased activity times, might affect their nutritional status and contribute to overweight and obesity (47, 48), low cognitive performance, and poor quality of life (49). The odds of overweight or obesity were nearly four times greater for those who gave up too easily on their healthy dietary habits (50).

Previous studies reported a statistically significant decrease in the daily consumption of carbonated drinks in the intervention group compared to control group (51, 52). However, different from previous studies, our study showed that national health lifestyles intervention on obesity did not exert great effects on students' practice, which could be partly attributed to the relatively short duration and a few uncontrolled covariates (student's healthy status, school education level and student's physical fitness, etc.), which might affect students' practice. It might also be attributed to changes in only one healthy habit in previous researches (51, 52). There was an urgent need to develop comprehensive intervention programs with a longer period to promote healthy lifestyles for all school students, in order to help them to maintain good nutritional status, better cognitive abilities, and great quality of life.

Because parents were of major importance regarding their children's nutrition and outdoor activities, parents should be actively involved in school-based obesity prevention efforts. A study showed that parental beliefs toward childhood obesity were the most important determinant of childhood obesity (45, 53). Notably, parents played an extremely influential role in supporting and managing practice that affect children's energy balance (diet, physical activity, etc.), which were integral to the intervention (54, 55). Previous study reported that, after an intervention program targeted to Mexican mothers of school-aged children, aiming at preventing childhood obesity, the mother's nutrition related knowledge significantly increased from baseline, and had positive significance for the prevention of childhood obesity (56). However, in this study, though there was no significant change in parental knowledge related to obesity, and parents were more likely to perform worse toward a few knowledges, an improvement in parents' beliefs and practice toward childhood obesity was observed. Although nutrition knowledge was necessary, it could be insufficient to promote health practice changes (57). The change on mothers' practices such as restricting the children's sugar intake and preparing breakfast for children could lead to the improvement of long-term outcomes in the children's nutrition status when the practice was maintained consistently over time. Though the existing literature suggested that parental self-efficacy and food environment was highly associated with children's healthy dietary practice (58), there were potential differences in the way families respond to obesity-related interventions. For example, in families with good practice and lifestyle, family members are able to adapt to internal and external demands for change easily, such as demands for a healthy lifestyle. In contrast, dysfunctional families could become more rigid in the face of change, making it difficult to adopt new approaches to diet and exercise (26, 59). Besides, parents highlighted that in case educational materials will be provided (e.g., leaflet, brochure), great efforts should be made to make these as attractive as possible. Therefore, the participation of parents had also been reported in other studies as a challenging aspect of childhood obesity interventions (60, 61). Still, strategies were required so that parents with limited availability could benefit from this type of interventions.

In addition to family interventions, the school environment was considered as an important intervention site for obesity prevention. In this study, the intervention had little impact on the knowledge and beliefs of the schools' administrators, but there was a significant improvement of school's practice or measures, such as arranging sports activities in the afternoon and sugar-sweetened beverages were not available in the school canteen. The combination of academic stress and competition between schools might have led to the results. A study involving 141 high school students aged 14 to 18 from Holon showed that school education about food and nutrition only amounted to 28.3% of the total (62). Therefore, schools also need to enhance health literacy education for students. From the perspective of public health, as children and adolescents spent most of their daytime at school, schools should take measures aiming at preventing obesity including restricting the marketing of unhealthy foods to children, improving school meals by setting binding quality standards, introducing a ban on the carrying and sale of sugary drinks to reduce the consumption of unhealthy foods, and increasing daily physical activity by providing more opportunities and times for outdoor activity (40, 63). These measures were increasingly being considered as mandatory for sustainable success in the fight against the obesity epidemic and were closely linked to the WHO Global Action Plan for the Prevention and Control of Non-communicable Diseases (64).

The strengths of this study were as follows. Firstly, we obtained data based on the national representative sample from seven provinces in China, and we focused on 6–18-year-old children and adolescents. Secondly, data were obtained on knowledge beliefs and practice among student, family, and school dimensions, allowing for a multifaceted analysis of the program's impact on childhood overweight and obesity. Besides, we included data from multiple districts and schools and conduct regular follow-up studies, allowing us to see the effects of the program over 6 months. In conclusion, this study considered the effects of childhood obesity interventions on individual, family and school environment. Some limitations should also be paid attention. Firstly, a questionnaire-based study cannot fundamentally explain the underlying reasons for the results. Secondly, the questionnaire survey could result in a possible trend of respondents toward compliance, which could favor positive outcomes to some extent. Thirdly, the lifestyle data was derived completed from a questionnaire, the dietary data recall of 7 days might not represent long-term dietary behaviors. The long-term dietary behavior might have other results to our current 7-day dietary data. Therefore, in future studies, the use of food tracking, such as photo tracking, could be used to accurately assess the frequency and intake of fruits and other dietary intakes of individuals. Fourthly, the content of knowledge, belief, and practice at the individual, family, and school levels were not comprehensive, and the information was limited, which cannot effectively and comprehensively reflect the real situation of students. We did not taken into account other possible correlated factors of obesity intervention, such as exposure to social media, social comparison, and psychological factors (65). Our results could only reflect part of the situation of the students, and there were still many questions that had not received feedback, which leads to one-sidedness in our results and biased differences in research conclusions. To get more real and comprehensive results, we need to consider all the content more comprehensively and systematically in our later research. However, trained project members interpreted all the questionnaires in detail, and appropriate guidance would be given by these project members as effectively as possible. The questionnaires would also be rechecked by 3% within 1 week for the same participants. Therefore, the quality of lifestyle information was guaranteed largely.

## Conclusion

In summary, this multi-center, school-based, 6-month obesity intervention with family and school participation in China significantly improved the obesity-related knowledge and beliefs among children and adolescents aged 6–18 years, but their lifestyle practice did not get satisfactory improvements, which could be an important reason for the limited effect of many international child obesity intervention projects. Besides, limited improvement in knowledge, beliefs, and certain practice were also observed among parents and schools. Further obesity related interventions with longer duration and more intensive interventions should be implemented to verify the conclusion. At the same time, the role of parents and schools' administrators in obesity intervention should still be strengthened to protect children and adolescents from being overweight or obesity.

## Data availability statement

The raw data supporting the conclusions of this article will be made available by the authors, without undue reservation.

## Ethics statement

The studies involving human participants were reviewed and approved by Peking University (IRB0000105213034). Written informed consent to participate in this study was provided by the participants' legal guardian/next of kin.

## Author contributions

XW and JL performed the data analysis. XW, JL, DG, QM, YL, TM, MC, and LC interpreted, wrote, and finalized the manuscript. YZ, YD, JY, YS, and JM participate in the reviewing and revising the manuscript. All authors contributed in conceiving, design of this study, read, and approved the final manuscript.

## Funding

This study was granted by the Research Special Fund for Public Welfare Industry of Health (201202010) and the National Natural Science Foundation of China (81673192) awarded to JM. Capital's Funds for Health Improvement and Research (2022-1G-4251 to YS), the Natural Science Foundation of Beijing (Grant No. 7222247 to YS).

## Conflict of interest

The authors declare that the research was conducted in the absence of any commercial or financial relationships that could be construed as a potential conflict of interest.

## Publisher's note

All claims expressed in this article are solely those of the authors and do not necessarily represent those of their affiliated organizations, or those of the publisher, the editors and the reviewers. Any product that may be evaluated in this article, or claim that may be made by its manufacturer, is not guaranteed or endorsed by the publisher.
