# Supplementary material for: Effectiveness of national multicentric school-based health lifestyles intervention among chinese children and adolescents on knowledge, belief, and practice toward obesity at individual, family and schools' levels
Source: Front Pediatr. 2022 Aug 18;10:917376. doi: 10.3389/fped.2022.917376 (PMC9433560; doi:10.3389/fped.2022.917376)
Supplement: Supplementary file 1 [file Data_Sheet_1.PDF]

Supplementary Material

Table S1. Effectiveness on the student's knowledge, belief, and practice (by Only-child / Non-only child).

Indicators	Time	Only-child		Non-only child		Total	
		Intervention	Control	Intervention	Control	Intervention	Control
Knowledge							
It is necessary to exercise every day. [yes]	Baseline (%)	90.18	90.11	89.29	88.95	89.90	89.73
	Post-intervention (%)	92.26	91.19	91.98	90.25	92.17	90.89
	Change (%)	2.08	1.08	2.69	1.30	2.27	1.16
	OR (95% CI)	**1.17 (1.04-1.31)**		**1.23 (1.05-1.44)**		**1.19 (1.08-1.30)**	
	p-values	**0.007**		**0.011**		**<0.001**	
It is healthier to drink plain boiled water than sugar-sweetened beverages. [yes]	Baseline (%)	84.94	85.55	83.99	83.75	84.64	84.96
	Post-intervention (%)	82.52	81.94	81.62	79.97	82.24	81.30
	Change (%)	-2.42	-3.61	-2.37	-3.78	-2.40	-3.66
	OR (95% CI)	**1.10 (1.01-1.20)**		1.11 0.98-1.25)		**1.10 (1.03-1.18)**	
	p-values	**0.024**		0.102		**0.006**	
The food at the bottom of the dietary pagoda should be eaten more. [yes]	Baseline (%)	35.87	35.67	29.23	26.78	33.83	32.79
	Post-intervention (%)	46.47	42.85	39.09	34.59	44.21	40.18
	Change (%)	10.60	7.18	9.86	7.81	10.38	7.39
	OR (95% CI)	**1.23 (1.14-1.33)**		1.11 (0.99-1.25)		**1.20 (1.12-1.27)**	
	p-values	**<0.001**		0.063		**<0.001**	
Obesity is bad for health. [yes]	Baseline (%)	81.57	79.12	78.36	75.28	80.59	77.88
	Post-intervention (%)	85.40	79.29	83.39	75.97	84.79	78.22
	Change (%)	3.83	0.17	5.03	0.69	4.20	0.34
	OR (95% CI)	**1.44 (1.32-1.58)**		**1.47 (1.30-1.67)**		**1.45 (1.35-1.56)**	
	p-values	**<0.001**		**<0.001**		**<0.001**	
Belief							
Believe you can achieve an ideal weight status through effort. [true]	Baseline (%)	68.28	67.91	65.65	66.44	67.47	67.44
	Post-intervention (%)	71.69	69.21	70.02	67.35	71.18	68.61
	Change (%)	3.41	1.30	4.37	0.91	3.71	1.17
	OR (95% CI)	**1.14(1.06-1.23)**		**1.22(1.10-1.36)**		**1.17 (1.10-1.24)**	
	p-values	**<0.001**		**<0.001**		**<0.001**	

Eat more fruits and vegetables and less high-energy snacks to lose weight. [true]	Baseline (%)	46.29	44.02	46.90	47.44	46.48	45.13
	Post-intervention (%)	52.75	48.77	53.38	52.12	52.94	49.86
	Change (%)	6.46	4.75	6.48	4.68	6.46	4.73
	OR (95% CI)	**1.11 (1.03-1.19)**		1.11 (1.00-1.23)		**1.11 (1.04-1.18)**	
	p-values	**0.007**		0.059		**0.001**	
Exercise to lose weight. [true]	Baseline (%)	49.43	48.47	49.14	50.89	49.34	49.26
	Post-intervention (%)	56.27	53.58	57.33	56.20	56.60	54.43
	Change (%)	6.84	5.11	8.19	5.31	7.26	5.17
	OR (95% CI)	**1.11 (1.03-1.19)**		**1.17 (1.06-1.21)**		**1.13 (1.06-1.20)**	
	p-values	**0.007**		**0.003**		**<0.001**	
Practice							
Fruit [daily intake≥3 servings/day]	Baseline (%)	8.71	7.75	8.03	7.45	8.50	7.65
	Post-intervention (%)	10.44	8.23	9.16	8.09	10.05	8.19
	Change (%)	1.73	0.48	1.13	0.64	1.55	0.54
	OR (95% CI)	**1.18 (1.05-1.33)**		1.07 (0.90-1.27)		**1.14 (1.04-1.26)**	
	p-values	**0.006**		0.474		**0.007**	
Sugar-sweetened beverages [daily intake=0 cups/week]	Baseline (%)	32.68	32.78	33.12	32.85	32.81	32.80
	Post-intervention (%)	36.10	36.37	39.30	36.36	37.09	36.37
	Change (%)	3.42	3.59	6.18	3.51	4.28	3.57
	OR (95% CI)	0.99 (0.92-1.07)		**1.17 (1.05-1.30)**		1.04 (0.98-1.11)	
	p-values	0.791		**0.005**		0.176	
Breakfast [frequency=7 days/week]	Baseline (%)	84.90	84.78	82.39	80.80	84.13	83.48
	Post-intervention (%)	86.09	85.11	84.80	80.89	85.69	83.74
	Change (%)	1.19	0.33	2.41	0.09	1.56	0.26
	OR (95% CI)	**1.12 (1.01-1.24)**		**1.28 (1.11-1.48)**		**1.17 (1.08-1.27)**	
	p-values	**0.040**		**0.001**		**<0.001**	
Milk [frequency=7 days/week]	Baseline (%)	45.65	45.50	35.67	37.95	42.57	43.05
	Post-intervention (%)	49.13	45.33	39.58	37.56	46.19	42.81
	Change (%)	3.48	-0.17	3.91	-0.39	3.62	-0.24
	OR (95% CI)	**1.25 (1.16-1.35)**		**1.31 (1.18-1.46)**		**1.27 (1.20-1.35)**	
	p-values	**< 0.001**		**< 0.001**		**< 0.001**	

Note: Model was adjusted for age, sex, province, and urban/rural area.

Table S2. Effectiveness on the student's knowledge, belief, and practice (by urban/rural area).

Indicators	Time	urban		rural		Total	
		Intervention	Control	Intervention	Control	Intervention	Control
Knowledge							
It is necessary to exercise every day. [yes]	Baseline (%)	89.94	89.89	89.85	89.49	89.90	89.73
	Post-intervention (%)	92.14	91.19	92.22	90.40	92.17	90.89
	Change (%)	2.20	1.30	2.37	0.91	2.27	1.16
	OR (95% CI)	**1.15 (1.02-1.29)**		**1.25 (1.08-1.45)**		**1.19 (1.08-1.30)**	
	p-values	**0.022**		**0.002**		**<0.001**	
It is healthier to drink plain boiled water than sugar-sweetened beverages. [yes]	Baseline (%)	84.79	85.31	84.42	84.42	84.64	84.96
	Post-intervention (%)	82.77	82.32	81.43	79.69	82.24	81.30
	Change (%)	-2.02	-2.99	-2.99	-4.73	-2.40	-3.66
	OR (95% CI)	1.08 (0.99-1.19)		**1.13 (1.02-1.26)**		**1.10 (1.03-1.18)**	
	p-values	0.081		**0.025**		**0.006**	
The food at the bottom of the dietary pagoda should be eaten more. [yes]	Baseline (%)	36.35	33.15	30.08	32.24	33.83	32.79
	Post-intervention (%)	46.60	42.04	40.64	37.29	44.21	40.18
	Change (%)	10.25	8.89	10.56	5.05	10.38	7.39
	OR (95% CI)	1.07 (0.99-1.16)		**1.42 (1.29-1.58)**		**1.20 (1.12-1.27)**	
	p-values	0.093		**<0.001**		**<0.001**	
Obesity is bad for health. [yes]	Baseline (%)	80.31	77.85	81.02	77.92	80.59	77.88
	Post-intervention (%)	84.18	78.43	85.71	77.89	84.79	78.22
	Change (%)	3.87	0.58	4.69	-0.03	4.20	0.34
	OR (95% CI)	**1.37 (1.25-1.52)**		**1.58 (1.40-1.77)**		**1.45 (1.35-1.56)**	
	p-values	**<0.001**		**<0.001**		**<0.001**	
Belief							
Believe you can achieve an ideal weight status through effort. [true]	Baseline (%)	67.71	67.24	67.11	66.75	67.47	67.44
	Post-intervention (%)	70.85	68.09	71.68	69.43	71.18	68.61
	Change (%)	3.14	0.85	4.57	2.68	3.71	1.17
	OR (95% CI)	**1.15 (1.07-1.24)**		**1.19(1.08-1.31)**		**1.17 (1.10-1.24)**	
	p-values	**<0.001**		**<0.001**		**<0.001**	

Eat more fruits and vegetables and less high-energy snacks to lose weight. [true]	Baseline (%)	46.23	44.52	46.85	46.08	46.48	45.13
	Post-intervention (%)	52.73	49.46	53.27	50.47	52.94	49.86
	Change (%)	6.50	4.94	6.42	4.39	6.46	4.73
	OR (95% CI)	**1.09 (1.01-1.18)**		**1.13 (1.02-1.24)**		**1.11 (1.04-1.18)**	
	p-values	**0.024**		**0.014**		**<0.001**	
Exercise to lose weight. [true]	Baseline (%)	49.34	48.49	49.35	50.45	49.34	49.26
	Post-intervention (%)	56.54	54.45	56.67	54.41	56.60	54.43
	Change (%)	7.20	5.96	7.32	3.96	7.26	5.17
	OR (95% CI)	1.07 (0.99-1.16)		**1.21 (1.10-1.33)**		**1.13 (1.06-1.20)**	
	p-values	0.069		**<0.001**		**<0.001**	
Practice							
Fruit [daily intake≥3 servings/day]	Baseline (%)	9.32	8.44	7.26	6.41	8.50	7.65
	Post-intervention (%)	10.70	8.87	9.07	7.11	10.05	8.19
	Change (%)	1.38	0.43	1.81	0.70	1.55	0.54
	OR (95% CI)	0.89 (0.82-0.96)		**1.63 (1.47-1.79)**		**1.14 (1.04-1.26)**	
	p-values	0.002		**<0.001**		**0.007**	
Sugar-sweetened beverages [daily intake=0 cups/week]	Baseline (%)	33.46	32.12	31.83	33.86	32.81	32.80
	Post-intervention (%)	35.75	36.64	39.11	35.94	37.09	36.37
	Change (%)	2.29	4.52	7.28	2.08	4.28	3.57
	OR (95% CI)	0.87 (0.80-0.94)		**1.36 (1.24-1.50)**		1.04 (0.98-1.11)	
	p-values	0.001		**<0.001**		0.175	
Breakfast [frequency=7 days/week]	Baseline (%)	85.28	83.82	82.37	82.95	84.13	83.48
	Post-intervention (%)	86.57	84.69	84.35	82.23	85.69	83.74
	Change (%)	1.29	0.87	1.98	-0.72	1.56	0.26
	OR (95% CI)	1.07 (0.96-1.20)		**1.32 (1.17-1.51)**		**1.17 (1.08-1.27)**	
	p-values	0.221		**<0.001**		**<0.001**	
Milk [frequency=7 days/week]	Baseline (%)	42.97	43.48	41.96	42.37	42.57	43.05
	Post-intervention (%)	45.86	42.71	46.69	42.96	46.19	42.81
	Change (%)	2.89	-0.77	4.73	0.59	3.62	-0.24
	OR (95% CI)	**1.26 (1.17-1.37)**		**1.29 (1.17-1.42)**		**1.27 (1.20-1.35)**	
	p-values	**< 0.001**		**< 0.001**		**< 0.001**	

Note: Model was adjusted for age, sex, province .

Table S3. Effectiveness on the parents' knowledge, belief, and practice (by maternal occupation)

Indicators	Time	Administrator, clerk and Professional		Business, Services and Others		Total	
		Intervention	Control	Intervention	Control	Intervention	Control
Knowledge							
It is necessary to exercise every day. [yes]	Baseline (%)	92.49	92.53	90.07	89.27	90.12	89.33
	Post-intervention (%)	93.29	92.22	90.58	90.33	90.77	90.43
	Change (%)	0.80	-0.31	0.51	1.06	0.65	1.10
	OR (95% CI)	1.22 (0.90-1.66)		1.02 (0.88-1.17)		0.95 (0.86-1.04)	
	p-values	0.193		0.806		0.243	
It is healthier to drink plain boiled water than sugar-sweetened beverages. [yes]	Baseline (%)	95.42	95.72	94.66	94.49	94.63	92.42
	Post-intervention (%)	92.78	92.52	92.17	91.81	92.05	91.79
	Change (%)	-2.64	-3.20	-2.49	-2.68	-2.58	-2.63
	OR (95% CI)	1.12 (0.81-1.55)		1.02 (0.89-1.16)		1.00 (0.89-1.11)	
	p-values	0.486		0.782		0.930	
The food at the bottom of the dietary pagoda should be eaten more. [yes]	Baseline (%)	48.40	49.92	32.43	30.98	34.22	32.66
	Post-intervention (%)	53.13	54.91	39.10	37.62	40.37	39.14
	Change (%)	4.73	4.99	6.67	6.64	6.15	6.48
	OR (95% CI)	0.99 (0.83-1.17)		1.00 (0.92-1.08)		0.98 (0.91-1.04)	
	p-values	0.865		0.918		0.440	
Obesity is bad for health. [yes]	Baseline (%)	92.20	91.51	87.59	86.69	87.91	86.77
	Post-intervention (%)	91.24	90.68	87.73	86.54	87.93	86.79
	Change (%)	-0.96	-0.83	0.14	-0.15	0.02	0.02
	OR (95% CI)	0.97 (0.73-1.29)		1.03 (0.93-1.15)		1.00 (0.92-1.09)	
	p-values	0.834		0.528		0.985	
Belief							
Don't buy drinks for children. [true]	Baseline (%)	46.61	47.19	43.90	45.34	44.04	45.24
	Post-intervention (%)	49.14	47.11	47.65	46.61	47.57	46.72
	Change (%)	2.53	-0.08	3.75	1.27	3.53	1.48
	OR (95% CI)	1.17 (0.98-1.39)		**1.15 (1.07-1.24)**		**1.12 (1.06-1.19)**	
	p-values	0.085		**<0.001**		**<0.001**	
Children take part in physical	Baseline (%)	74.89	77.80	75.06	76.87	75.15	77.03
	Post-intervention (%)	79.25	78.92	78.88	79.18	78.95	78.73

exercise with their classmates. [true]	Change (%)	4.36	1.12	3.82	2.31	3.80	1.70
	OR (95% CI)	**1.27 (1.04-1.55)**		**1.11 (1.02-1.21)**		**1.16 (1.08-1.24)**	
	p-values	**0.019**		**0.015**		**<0.001**	
Set aside time every day for children to exercise. [true]	Baseline (%)	79.85	83.53	80.27	82.19	80.36	82.31
	Post-intervention (%)	83.08	86.06	83.88	84.34	83.77	84.31
	Change (%)	3.23	2.53	3.61	2.15	3.41	2.00
	OR (95% CI)	1.03 (0.83-1.29)		**1.12 (1.02-1.23)**		**1.12 (1.04-1.21)**	
	p-values	0.781		**0.013**		**0.004**	
Look at the nutrition label when shopping in the supermarket. [true]	Baseline (%)	73.95	72.70	70.58	71.61	71.17	71.93
	Post-intervention (%)	76.54	74.60	74.76	74.27	74.85	74.20
	Change (%)	2.59	1.90	4.18	2.66	3.68	2.27
	OR (95% CI)	1.05 (0.87-1.28)		**1.11 (1.02-1.20)**		**1.10 (1.03-1.18)**	
	p-values	0.589		**0.013**		**0.006**	
Drinks are not always available at home. [true]	Baseline (%)	68.77	70.61	68.45	68.91	68.41	69.22
	Post-intervention (%)	71.33	71.46	69.82	69.49	69.87	69.88
	Change (%)	2.56	0.85	1.37	0.58	1.46	0.66
	OR (95% CI)	1.13 (0.93-1.37)		1.05 (0.97-1.14)		1.05(0.99-1.13)	
	p-values	0.223		0.196		0.118	
Practice							
Fruit [daily intake≥3 servings/day]	Baseline (%)	6.80	4.89	6.03	5.43	6.34	5.58
	Post-intervention (%)	6.66	6.66	6.84	6.42	6.96	6.60
	Change (%)	-0.14	1.77	0.81	0.99	0.62	1.02
	OR (95% CI)	**0.66 (0.47-0.92)**		0.95 (0.83-1.09)		0.92 (0.82-1.03)	
	p-values	**0.014**		0.504		0.130	
Sugar-sweetened beverages [daily intake≤4 cups/week]	Baseline (%)	93.74	94.48	91.80	92.56	91.91	92.65
	Post-intervention (%)	93.77	93.87	92.45	92.43	92.54	92.53
	Change (%)	0.03	-0.61	0.65	-0.13	0.63	-0.12
	OR (95% CI)	1.15 (0.82-1.61)		1.13 (1.00-1.29)		**1.13 (1.02-1.26)**	
	p-values	0.420		0.051		**0.025**	
Breakfast [frequency=7 days/week]	Baseline (%)	89.66	90.68	84.31	85.62	84.97	85.96
	Post-intervention (%)	91.70	90.68	86.92	86.06	87.57	86.64
	Change (%)	2.04	0.00	2.61	0.44	2.60	0.68
	OR (95% CI)	**1.34 (1.02-1.76)**		**1.25 (1.14-1.39)**		**1.23 (1.13-1.34)**	
	p-values	**0.037**		**<0.001**		**<0.001**	
	Baseline (%)	32.84	30.93	27.45	26.79	28.10	27.63

Milk [frequency=7 days/week]	Post-intervention (%)	36.88	33.23	31.30	28.86	31.71	29.66
	Change (%)	4.04	2.30	3.85	2.07	3.61	2.03
	OR (95% CI)	1.10 (0.92-1.32)		**1.12 (1.03-1.21)**		**1.10 (1.03-1.18)**	
	p-values	0.287		**0.006**		**0.003**	

Note: Model was adjusted for age, sex, province, and urban/rural area.

Table S4. Effectiveness on the parents' knowledge, belief, and practice (by monthly household income).

Indicators	Time	≥2000		< 2000		Total	
		Intervention	Control	Intervention	Control	Intervention	Control
Knowledge							
It is necessary to exercise every day. [yes]	Baseline (%)	91.59	91.74	90.17	89.35	90.12	89.33
	Post-intervention (%)	92.26	92.34	90.70	90.24	90.77	90.43
	Change (%)	0.67	0.60	0.53	0.89	0.65	1.10
	OR (95% CI)	1.01 (0.79-1.30)		1.02 (0.88-1.17)		0.95 (0.86-1.04)	
	p-values	0.930		0.806		0.243	
It is healthier to drink plain boiled water than sugar-sweetened beverages. [yes]	Baseline (%)	95.31	95.53	94.64	94.31	94.63	94.42
	Post-intervention (%)	93.31	92.20	92.07	91.87	92.05	91.79
	Change (%)	-2.00	-3.33	-2.57	-2.44	-2.58	-2.63
	OR (95% CI)	1.26 (0.95-1.67)		0.96 (0.84-1.11)		1.00 (0.89-1.11)	
	p-values	0.102		0.599		0.930	
The food at the bottom of the dietary pagoda should be eaten more. [yes]	Baseline (%)	44.35	43.35	33.03	31.83	34.22	32.66
	Post-intervention (%)	50.31	49.12	39.53	38.20	40.37	39.14
	Change (%)	5.96	5.77	6.50	6.37	6.15	6.48
	OR (95% CI)	1.01 (0.87-1.17)		1.00 (0.92-1.09)		0.98 (0.91-1.04)	
	p-values	0.900		0.958		0.440	
Obesity is bad for health. [yes]	Baseline (%)	91.12	90.53	87.90	86.90	87.91	86.77
	Post-intervention (%)	90.45	89.72	88.06	86.71	87.93	86.79
	Change (%)	-0.67	-0.81	0.16	-0.19	0.02	0.02
	OR (95% CI)	1.02 (0.80-1.29)		1.04 (0.93-1.17)		1.00 (0.92-1.09)	
	p-values	0.887		0.479		0.985	
Belief							

Don't buy drinks for children. [true]	Baseline (%)	44.92	45.79	44.02	44.91	44.04	45.24
	Post-intervention (%)	48.61	46.25	47.58	46.32	47.57	46.72
	Change (%)	3.69	0.46	3.56	1.41	3.53	1.48
	OR (95% CI)	**1.10 (1.00-1.21)**		**1.22 (1.01-1.48)**		**1.12 (1.06-1.19)**	
	p-values	**0.044**		**0.041**		**<0.001**	
Children take part in physical exercise with their classmates. [true]	Baseline (%)	75.56	77.46	75.14	76.80	75.15	77.03
	Post-intervention (%)	79.35	79.02	78.81	78.96	78.95	78.73
	Change (%)	3.79	1.56	3.67	2.16	3.80	1.70
	OR (95% CI)	1.10 (0.99-1.22)		**1.45 (1.15-1.83)**		**1.16 (1.08-1.24)**	
	p-values	0.085		**0.001**		**<0.001**	
Set aside time every day for children to exercise. [true]	Baseline (%)	80.54	82.67	80.12	82.32	80.36	82.31
	Post-intervention (%)	83.77	84.74	83.43	84.30	83.77	84.31
	Change (%)	3.23	2.07	3.31	1.98	3.41	2.00
	OR (95% CI)	1.07 (0.95-1.21)		**1.32 (1.03-1.70)**		**1.12 (1.04-1.21)**	
	p-values	0.239		**0.027**		**0.004**	
Look at the nutrition label when shopping in the supermarket. [true]	Baseline (%)	73.81	72.65	70.35	71.82	71.17	71.93
	Post-intervention (%)	76.04	75.79	74.72	73.67	74.85	74.20
	Change (%)	72.65	70.35	4.37	1.85	3.68	2.27
	OR (95% CI)	**1.09 (0.98-1.21)**		1.21 (0.98-1.49)		**1.10 (1.03-1.18)**	
	p-values	**0.114**		0.081		**0.006**	
Drinks are not always available at home. [true]	Baseline (%)	68.73	69.83	68.00	68.69	68.41	69.22
	Post-intervention (%)	70.61	70.14	69.48	69.03	69.87	69.88
	Change (%)	1.88	0.31	1.48	0.34	1.46	0.66
	OR (95% CI)	**1.14 (1.04-1.26)**		1.05 (0.85-1.31)		1.05(0.99-1.13)	
	p-values	**0.008**		0.653		0.118	
Practice							
Fruit [daily intake≥3 servings/day]	Baseline (%)	6.50	5.46	6.01	5.39	6.34	5.58
	Post-intervention (%)	6.34	6.28	7.00	6.47	6.96	6.60
	Change (%)	-0.16	0.82	0.99	1.08	0.62	1.02
	OR (95% CI)	0.82 (0.62-1.09)		0.97 (0.83-1.12)		0.92 (0.82-1.03)	
	p-values	0.168		0.643		0.130	
Sugar-sweetened beverages [daily	Baseline (%)	93.85	93.78	91.51	92.56	91.91	92.65
	Post-intervention (%)	93.56	93.48	92.48	92.40	92.54	92.53
	Change (%)	-0.29	-0.30	0.97	-0.16	0.63	-0.12

intake≤4 cups/week]	OR (95% CI)	1.00 (0.75-1.33)		**1.20 (1.04-1.37)**		**1.13 (1.02-1.26)**	
	p-values	0.992		**0.010**		**0.025**	
Breakfast [frequency=7 days/week]	Baseline (%)	88.58	87.99	84.23	85.84	84.97	85.96
	Post-intervention (%)	90.53	89.14	87.00	86.10	87.57	86.64
	Change (%)	1.95	1.15	2.77	0.26	2.60	0.68
	OR (95% CI)	1.12 (0.90-1.41)		**1.30 (1.17-1.45)**		**1.23 (1.13-1.34)**	
	p-values	0.307		**<0.001**		**<0.001**	
Milk [frequency=7 days/week]	Baseline (%)	32.16	29.59	27.60	27.17	28.10	27.63
	Post-intervention (%)	36.07	31.22	31.64	29.25	31.71	29.66
	Change (%)	3.91	1.63	4.04	2.08	3.61	2.03
	OR (95% CI)	1.14 (0.98-1.34)		**1.13 (1.04-1.23)**		**1.10 (1.03-1.18)**	
	p-values	0.095		**0.005**		**0.003**	

Note: Model was adjusted for age, sex, province, and urban/rural area. Monthly household income (RMB)

Table S5. Effectiveness on the parents' knowledge, belief, and practice (by urban/rural area).

Indicators	Time	urban		rural		Total	
		Intervention	Control	Intervention	Control	Intervention	Control
Knowledge							
It is necessary to exercise every day. [yes]	Baseline (%)	90.52	90.35	89.51	87.81	90.12	89.33
	Post-intervention (%)	90.84	91.40	90.68	89.00	90.77	90.43
	Change (%)	0.32	1.05	1.17	1.19	0.65	1.10
	OR (95% CI)	0.90 (0.79-1.02)		1.02 (0.88-1.17)		0.95 (0.86-1.04)	
	p-values	0.090		0.806		0.243	
It is healthier to drink plain boiled water than sugar-sweetened beverages.[yes]	Baseline (%)	94.65	94.32	94.60	94.57	94.63	94.42
	Post-intervention (%)	92.24	91.02	91.77	92.99	92.05	91.79
	Change (%)	-2.41	-3.30	-2.83	-1.58	-2.58	-2.63
	OR (95% CI)	1.11 (0.97-1.28)		0.83 (0.70-0.98)		1.00 (0.89-1.11)	
	p-values	0.130		0.030		0.930	
The food at the bottom of the dietary pagoda should be eaten more. [yes]	Baseline (%)	36.28	34.64	31.19	29.59	34.22	32.66
	Post-intervention (%)	42.86	41.19	36.72	35.96	40.37	39.14
	Change (%)	6.58	6.55	5.53	6.37	6.15	6.48
	OR (95% CI)	1.06 (0.92-1.08)		0.94 (0.85-1.04)		0.98 (0.91-1.04)	
	p-values	0.936		0.238		0.440	
Obesity is bad for health. [yes]	Baseline (%)	87.82	86.47	88.04	87.24	87.91	86.77
	Post-intervention (%)	88.07	85.97	87.72	88.09	87.93	86.79
	Change (%)	0.25	-0.50	-0.32	0.85	0.02	0.02
	OR (95% CI)	1.09 (0.97-1.22)		0.87 (0.76-1.01)		1.00 (0.92-1.09)	

	p-values	0.135		0.060		0.985	
Belief							
Don't buy drinks for children. [true]	Baseline (%)	44.19	45.82	43.82	44.33	44.04	45.24
	Post-intervention (%)	47.56	46.88	47.60	46.46	47.57	46.72
	Change (%)	3.37	1.06	3.78	2.13	3.53	1.48
	OR (95% CI)	**1.14 (1.05-1.25)**		**1.09 (0.99-1.20)**		**1.12 (1.06-1.19)**	
	p-values	**0.001**		**0.070**		**<0.001**	
Children take part in physical exercise with their classmates. [true]	Baseline (%)	73.92	77.02	76.97	77.06	75.15	77.03
	Post-intervention (%)	77.71	79.12	80.79	78.11	78.95	78.73
	Change (%)	3.79	2.10	3.82	1.05	3.80	1.70
	OR (95% CI)	**1.12 (1.02-1.22)**		**1.23 (1.10-1.38)**		**1.16 (1.08-1.24)**	
	p-values	**0.016**		**<0.001**		**<0.001**	
Set aside time every day for children to exercise. [true]	Baseline (%)	79.92	83.18	81.01	80.94	80.36	82.31
	Post-intervention (%)	83.50	85.70	84.16	82.12	83.77	84.31
	Change (%)	3.58	2.52	3.15	1.18	3.41	2.00
	OR (95% CI)	1.07 (0.97-1.18)		**1.19 (1.05-1.34)**		**1.12 (1.03-1.21)**	
	p-values	0.200		**0.005**		**0.004**	
Look at the nutrition label when shopping in the supermarket. [true]	Baseline (%)	72.11	71.98	69.77	71.85	71.17	71.93
	Post-intervention (%)	75.57	74.78	73.78	73.30	74.85	74.20
	Change (%)	3.46	2.80	4.01	1.45	3.68	2.27
	OR (95% CI)	**1.05 (0.96-1.15)**		**1.18 (1.06-1.31)**		**1.10 (1.03-1.18)**	
	p-values	**0.279**		**0.002**		**0.006**	
Drinks are not always available at home. [true]	Baseline (%)	68.11	68.85	68.86	69.80	68.41	69.22
	Post-intervention (%)	69.78	69.65	70.00	70.23	69.87	69.88
	Change (%)	1.67	0.80	1.14	0.43	1.46	0.66
	OR (95% CI)	1.06 (0.97-1.15)		1.05 (0.94-1.16)		1.05 (0.99-1.13)	
	p-values	0.178		0.393		0.118	
Practice							
Fruit [daily intake≥3 servings/day]	Baseline (%)	6.11	5.83	6.68	5.18	6.34	5.58
	Post-intervention (%)	7.15	6.61	6.67	6.57	6.96	6.60
	Change (%)	1.04	0.78	-0.01	1.39	0.62	1.02
	OR (95% CI)	1.04 (0.90-1.21)		0.76 (0.63-0.91)		0.92 (0.82-1.03)	
	p-values	0.590		0.003		0.130	
Sugar-sweetened beverages [daily intake≤4 cups/week]	Baseline (%)	91.97	92.64	91.81	92.65	91.91	92.65
	Post-intervention (%)	92.45	92.36	92.67	92.78	92.54	92.53
	Change (%)	0.48	-0.28	0.86	0.13	0.63	-0.12
	OR (95% CI)	1.13 (0.99-1.30)		**1.13 (0.95-1.33)**		**1.13 (1.02-1.26)**	
	p-values	0.079		**0.175**		**0.025**	
Breakfast [frequency=7 days/week]	Baseline (%)	84.79	86.40	85.23	85.27	84.97	85.96
	Post-intervention (%)	87.75	86.78	87.29	86.41	87.57	86.64
	Change (%)	2.96	0.38	2.06	1.14	2.60	0.68
	OR (95% CI)	**1.33(1.19-1.49)**		**1.10 (0.97-1.26)**		**1.23 (1.13-1.34)**	

	p-values	**<0.001**		**0.140**		**<0.001**	
Milk [frequency=7 days/week]	Baseline (%)	28.13	27.35	28.06	28.07	28.10	27.63
	Post-intervention (%)	32.55	28.42	30.46	31.58	31.71	29.66
	Change (%)	4.42	1.07	2.40	3.51	3.61	2.03
	OR (95% CI)	**1.24 (1.13-1.35)**		0.93 (0.84-1.04)		**1.10 (1.03-1.18)**	
	p-values	**<0.001**		0.195		**0.003**	

Note: Model was adjusted for age, sex, province .

Note: Model was adjusted for sex, province and urban/rural area.

Table S6. Effectiveness on the school's knowledge, belief, and practice (by Only-child / Non-only child).

Indicators	Time	Only child		Non-only child		Total	
		Intervention	Control	Intervention	Control	Intervention	Control
Knowledge							
Teaching activities crowd out break exercises or extracurricular sports activities. [yes]	Baseline (%)	72.89	73.49	73.15	74.89	72.97	73.95
	Post-intervention (%)	67.03	68.02	68.18	69.02	67.38	68.34
	Change (%)	-5.86	-5.47	-4.97	-5.87	-5.59	-5.61
	OR (95% CI)	0.98 (0.91-1.06)		1.06 (0.95-1.19)		1.01 (0.95-1.07)	
	p-values	0.629		0.270		0.830	
Belief							
The school arranges sports activities in the afternoon[frequency≥30min day/week. [true]	Baseline (%)	8.16	7.36	8.28	9.28	8.20	7.96
	Post-intervention (%)	9.70	9.27	11.18	11.64	10.16	10.03
	Change (%)	1.54	1.91	2.90	2.36	1.96	2.07
	OR (95% CI)	0.93 (0.79-1.09)		1.10 (0.88-1.38)		0.98 (0.86-1.11)	
	p-values	0.348		0.406		0.717	
Practice							
The school arranges sports activities in the afternoon	Baseline (%)	61.41	64.21	60.83	63.61	61.23	64.02
	Post-intervention (%)	68.25	69.08	68.14	68.50	68.21	68.89
	Change (%)	6.84	4.87	7.31	4.89	6.98	4.87
	OR (95% CI)	**1.11 (1.03-1.19)**		**1.14 (1.02-1.27)**		**1.12 (1.05-1.19)**	
	p-values	**0.005**		**0.018**		**<0.001**	
Sugar-sweetened beverages are not available in the school canteen	Baseline (%)	48.67	53.30	45.05	47.57	47.56	51.45
	Post-intervention (%)	47.15	49.38	44.32	42.55	46.28	47.17
	Change (%)	-1.52	-3.92	-0.73	-5.02	-1.28	-4.28

	OR (95% CI)	**1.27 (1.15-1.40)**	**1.48 (1.29-1.69)**	**1.33 (1.23-1.44)**
	p-values	**<0.001**	**<0.001**	**<0.001**

Note: Model was adjusted for age, sex, province, and urban/rural area.

Table S7. Effectiveness on the school's knowledge, attitude, and behavior (by parental education).

Indicators	Time	High school or above		Below high school		Total	
		Intervention	Control	Intervention	Control	Intervention	Control
Knowledge							
Teaching activities crowd out break exercises or extracurricular sports activities. [yes]	Baseline (%)	72.83	74.82	73.49	74.59	72.97	73.95
	Post-intervention (%)	66.80	69.20	68.56	69.34	67.38	68.34
	Change (%)	-6.03	-5.62	-4.93	-5.25	-5.59	-5.61
	OR (95% CI)	0.99 (0.91-1.07)		1.02 (0.92-1.14)		1.01 (0.95-1.07)	
	p-values	0.793		0.662		0.830	
Belief							
The school arranges sports activities in the afternoon[frequency≥30min day/week. [true]	Baseline (%)	7.77	6.85	8.67	10.40	8.20	7.96
	Post-intervention (%)	9.50	8.19	11.89	13.46	10.16	10.03
	Change (%)	1.73	1.34	3.22	3.06	1.96	2.07
	OR (95% CI)	1.05 (0.89-1.30)		1.06 (0.85-1.31)		0.98 (0.86-1.11)	
	p-values	0.554		0.621		0.717	
Practice							
The school arranges sports activities in the afternoon	Baseline (%)	63.31	65.54	60.16	63.26	61.23	64.02
	Post-intervention (%)	67.79	70.76	70.45	67.81	68.21	68.89
	Change (%)	4.48	5.22	10.29	4.55	6.98	4.87
	OR (95% CI)	0.95 (0.88-1.03)		**1.39 (1.25-1.55)**		**1.12(1.05-1.19)**	
	p-values	0.199		**<0.001**		**<0.001**	
Sugar-sweetened beverages are not available in the school canteen	Baseline (%)	52.55	58.61	44.44	45.69	47.56	51.45
	Post-intervention (%)	50.97	53.91	43.23	41.03	46.28	47.17
	Change (%)	-1.58	-4.70	-1.21	-4.66	-1.28	-4.28
	OR (95% CI)	**1.34 (1.21-1.49)**		**1.38 (1.21-1.58)**		**1.33 (1.23-1.44)**	
	p-values	**<0.001**		**<0.001**		**<0.001**	

Note: Model was adjusted for age, sex, province, and urban/rural area. High school or above: Either the father or the mother is high school or above. Below high school: Both father and mother have below high school education.

Table S8. Effectiveness on the school's knowledge, belief, and practice (by maternal occupation).

Indicators	Time	Administrator, clerk and Professional		Business, Services and Others		Total	
		Intervention	Control	Intervention	Control	Intervention	Control
Knowledge							
Teaching activities crowd out break exercises or extracurricular sports activities. [yes]	Baseline (%)	72.45	75.16	72.98	74.71	72.97	73.95
	Post-intervention (%)	65.58	68.99	67.80	69.19	67.38	68.34
	Change (%)	-6.87	-6.17	-5.18	-5.52	-5.59	-5.61
	OR (95% CI)	0.97 (0.81-1.17)		1.03 (0.95-1.11)		1.01 (0.95-1.07)	
	p-values	0.803		0.461		0.830	
Belief							
The school arranges sports activities in the afternoon[frequency≥30min day/week. [true]	Baseline (%)	7.93	7.38	8.06	7.73	8.20	7.96
	Post-intervention (%)	10.03	7.60	10.16	10.09	10.16	10.03
	Change (%)	2.10	0.22	2.10	2.36	1.96	2.07
	OR (95% CI)	1.42 (0.97-2.08)		0.94 (0.80-1.11)		0.98 (0.86-1.11)	
	p-values	0.068		0.470		0.717	
Practice							
The school arranges sports activities in the afternoon	Baseline (%)	63.06	66.56	62.07	64.04	61.23	64.02
	Post-intervention (%)	67.32	69.98	68.61	69.58	68.21	68.89
	Change (%)	4.26	3.42	6.54	5.54	6.98	4.87
	OR (95% CI)	1.04 (0.87-1.24)		1.05 (0.97-1.13)		**1.12 (1.05-1.19)**	
	p-values	0.655		0.201		**<0.001**	
Sugar-sweetened beverages are not available in the school canteen	Baseline (%)	51.21	58.51	48.94	52.40	47.56	51.45
	Post-intervention (%)	48.38	54.10	47.72	47.48	46.28	47.17
	Change (%)	-2.83	-4.41	-1.22	-4.92	-1.28	-4.28
	OR (95% CI)	1.15 (0.91-1.43)		**1.42 (1.29-1.57)**		**1.33 (1.23-1.44)**	
	p-values	0.236		**<0.001**		**<0.001**	

Note: Model was adjusted for age, sex, province, and urban/rural area.

Table S9. Effectiveness on the school's knowledge, belief, and practice (by monthly household income).

Indicators	Time	≥2000		< 2000		Total	
		Intervention	Control	Intervention	Control	Intervention	Control
Knowledge							
Teaching activities crowd out break exercises or extracurricular sports activities. [yes]	Baseline (%)	74.37	75.32	75.26	73.67	72.97	73.95
	Post-intervention (%)	68.56	70.23	69.85	69.31	67.38	68.34
	Change (%)	-5.81	-5.09	-5.41	-4.36	-5.59	-5.61
	OR (95% CI)	0.96 (0.87-1.07)		0.93 (0.75-1.16)		1.01 (0.95-1.07)	
	p-values	0.453		0.521		0.835	
Belief							
The school arranges sports activities in the afternoon [frequency≥30min day/week. [true]	Baseline (%)	7.77	7.44	9.71	12.91	8.20	7.96
	Post-intervention (%)	10.34	9.91	14.30	14.21	10.16	10.03
	Change (%)	2.57	2.47	4.59	1.30	1.96	2.07
	OR (95% CI)	1.01 (0.82-1.25)		1.46 (0.96-2.24)		0.98 (0.86-1.11)	
	p-values	0.934		0.08		**0.717**	
Practice							
The school arranges sports activities in the afternoon	Baseline (%)	62.24	63.07	60.95	64.68	61.23	64.02
	Post-intervention (%)	68.61	70.24	72.43	70.24	68.21	68.89
	Change (%)	6.37	7.17	11.48	5.56	6.98	4.87
	OR (95% CI)	0.95 (0.86-1.05)		**1.41 (1.14-1.75)**		1.12 (1.05-1.19)	
	p-values	0.305		**0.002**		**<0.001**	
Sugar-sweetened beverages are not available in the school canteen	Baseline (%)	44.79	52.30	47.68	43.94	47.56	51.45
	Post-intervention (%)	42.95	47.19	47.16	38.49	46.28	47.17
	Change (%)	-1.84	-5.11	-0.52	-5.45	-1.28	-4.28
	OR (95% CI)	**1.35 (1.19-1.53)**		**1.63 (1.24-2.15)**		**1.33 (1.23-1.44)**	
	p-values	**<0.001**		**<0.001**		**<0.001**	

Note: Model was adjusted for age, sex, province, and urban/rural area. Monthly household income (RMB)

Table S10. Effectiveness on the school's knowledge, belief, and practice (by urban/rural area).

Indicators	Time	urban		rural		Total	
		Intervention	Control	Intervention	Control	Intervention	Control
Knowledge							
Teaching activities crowd out break exercises or extracurricular sports activities. [yes]	Baseline (%)	73.19	74.91	72.63	72.42	72.97	73.95
	Post-intervention (%)	66.58	68.83	68.58	67.56	67.38	68.34
	Change (%)	-6.61	-6.08	-4.05	-4.86	-5.59	-5.61
	OR (95% CI)	0.98 (0.91-1.06)		1.05 (0.95-1.15)		1.01 (0.95-1.07)	
	p-values	0.661		0.376		0.832	
Belief							
The school arranges sports activities in the afternoon [frequency≥30min day/week. [true]	Baseline (%)	8.32	6.84	8.00	9.78	8.20	7.96
	Post-intervention (%)	10.89	8.60	9.20	12.31	10.16	10.03
	Change (%)	2.57	1.76	1.20	2.53	1.96	2.07
	OR (95% CI)	1.10 (0.93-1.30)		0.85 (0.70-1.05)		0.98 (0.86-1.12)	
	p-values	0.262		0.127		0.701	
Practice							
The school arranges sports activities in the afternoon	Baseline (%)	59.91	62.15	63.23	67.02	61.23	64.02
	Post-intervention (%)	64.83	69.01	73.32	68.70	68.21	68.89
	Change (%)	4.92	6.86	10.09	1.68	6.98	4.87
	OR (95% CI)	0.90 (0.79-1.02)		1.02 (0.88-1.17)		0.95 (0.86-1.04)	
	p-values	0.090		0.806		0.243	
Sugar-sweetened beverages are not available in the school canteen	Baseline (%)	42.10	47.94	55.82	57.08	47.56	51.45
	Post-intervention (%)	39.62	43.33	56.36	53.35	46.28	47.17
	Change (%)	-2.48	-4.61	0.54	-3.73	-1.28	-4.28
	OR (95% CI)	**1.21 (1.09-1.34)**		**1.48 (1.31-1.67)**		**1.33 (1.23-1.44)**	
	p-values	**<0.001**		**<0.001**		**<0.001**	

Note: Model was adjusted for age, sex, province .

Table S11. Effectiveness on the student's knowledge, belief, and practice (by maternal occupation).

Indicators	Time	Administrator, clerk and Professional		Business, Services and Others		Total	
		Intervention	Control	Intervention	Control	Intervention	Control
Knowledge							
It is necessary to exercise every day. [yes]	Baseline (%)	91.47	91.73	90.04	90.03	89.90	89.73
	Post-intervention (%)	93.20	93.25	92.45	90.86	92.17	90.89
	Change (%)	1.73	1.52	2.41	0.83	2.27	1.16
	OR (95% CI)	1.03 (0.78-1.39)		**1.27 (1.14-1.43)**		**1.19 (1.08-1.30)**	
	p-values	0.830		**<0.001**		**<0.001**	
It is healthier to drink plain boiled water than sugar-sweetened beverages. [yes]	Baseline (%)	85.60	86.13	84.67	84.88	84.64	84.96
	Post-intervention (%)	84.22	82.57	82.32	81.13	82.24	81.30
	Change (%)	-1.38	-3.56	-2.35	-3.75	-2.40	-3.66
	OR (95% CI)	1.20 (0.97-1.48)		**1.11 (1.02-1.21)**		**1.10 (1.03-1.18)**	
	p-values	0.089		**0.015**		**0.006**	
The food at the bottom of the dietary pagoda should be eaten more. [yes]	Baseline (%)	45.57	47.96	33.08	31.48	33.83	32.79
	Post-intervention (%)	55.16	54.44	43.26	39.50	44.21	40.18
	Change (%)	9.59	6.48	10.18	8.02	10.38	7.39
	OR (95% CI)	**1.21 (1.01-1.45)**		**1.13 (1.05-1.23)**		**1.20 (1.12-1.27)**	
	p-values	**0.036**		**0.002**		**<0.001**	
Obesity is bad for health. [yes]	Baseline (%)	84.20	82.18	80.85	78.40	80.59	77.88
	Post-intervention (%)	87.40	80.94	85.27	78.83	84.79	78.22
	Change (%)	3.20	-1.24	4.42	0.43	4.20	0.34
	OR (95% CI)	**1.62 (1.28-2.04)**		**1.48 (1.35-1.62)**		**1.45 (1.35-1.56)**	
	p-values	**<0.001**		**<0.001**		**<0.001**	
Belief							
Believe you can achieve an ideal weight status through effort. [true]	Baseline (%)	70.93	72.11	67.66	67.83	67.47	67.44
	Post-intervention (%)	73.77	71.85	71.25	68.84	71.18	68.61
	Change (%)	2.84	-0.26	3.59	1.01	3.71	1.17
	OR (95% CI)	**1.22(1.02-1.46)**		**1.17(1.08-1.26)**		**1.17 (1.10-1.24)**	
	p-values	**0.032**		**<0.001**		**<0.001**	
Eat more fruits and	Baseline (%)	44.38	43.29	47.38	45.97	46.48	45.13

vegetables and less high-energy snacks to lose weight. [true]	Post-intervention (%)	53.10	48.04	53.19	50.85	52.94	49.86
	Change (%)	8.72	4.75	5.81	4.88	6.46	4.73
	OR (95% CI)	**1.28 (1.07-1.54)**		1.06 (0.98-1.14)		**1.11 (1.04-1.18)**	
	p-values	**0.008**		0.149		**0.001**	
Exercise to lose weight. [true]	Baseline (%)	49.22	49.04	49.66	49.73	49.34	49.26
	Post-intervention (%)	57.69	54.11	56.76	55.06	56.60	54.43
	Change (%)	8.47	5.07	7.10	5.33	7.26	5.17
	OR (95% CI)	**1.21 (1.02-1.47)**		**1.11 (1.03-1.19)**		**1.13 (1.06-1.20)**	
	p-values	**0.026**		**0.007**		**<0.001**	
Practice							
Fruit [daily intake≥3 servings/day]	Baseline (%)	9.86	8.34	8.23	7.55	8.50	7.65
	Post-intervention (%)	11.15	9.93	9.82	8.11	10.05	8.19
	Change (%)	1.29	1.59	1.59	0.56	1.55	0.54
	OR (95% CI)	0.94 (0.70-1.24)		**1.15 (1.02-1.30)**		**1.14 (1.04-1.26)**	
	p-values	0.643		**0.024**		**0.007**	
Sugar-sweetened beverages [daily intake=0 cups/week]	Baseline (%)	36.93	37.02	32.50	32.13	32.81	32.80
	Post-intervention (%)	37.88	40.51	37.27	36.11	37.09	36.37
	Change (%)	0.95	3.49	4.77	3.98	4.28	3.57
	OR (95% CI)	0.86 (0.72-1.02)		1.05 (0.97-1.13)		1.04 (0.98-1.11)	
	p-values	0.094		0.238		0.176	
Breakfast [frequency=7 days/week]	Baseline (%)	90.42	91.59	84.97	84.03	84.13	83.48
	Post-intervention (%)	90.16	91.21	86.64	84.20	85.69	83.74
	Change (%)	-0.26	-0.38	1.67	0.17	1.56	0.26
	OR (95% CI)	1.03 (0.76-1.39)		**1.20 (1.08-1.33)**		**1.17 (1.08-1.27)**	
	p-values	0.845		**0.001**		**<0.001**	
Milk [frequency=7 days/week]	Baseline (%)	49.31	47.63	41.87	42.80	42.57	43.05
	Post-intervention (%)	46.31	46.41	45.39	41.98	46.19	42.81
	Change (%)	-3.00	-1.22	3.52	-0.82	3.62	-0.24
	OR (95% CI)	1.12 (0.94-1.35)		**1.30 (1.21-1.41)**		**1.27 (1.20-1.35)**	
	p-values	0.209		**< 0.001**		**< 0.001**	

Note: Model was adjusted for age, sex, province, and urban/rural area.

Table S12. Effectiveness on the student's knowledge, attitude, and behavior (by parental education).

Indicators	Time	High school or above		Below high school		Total	
		Intervention	Control	Intervention	Control	Intervention	Control
Knowledge							
It is necessary to exercise every day. [yes]	Baseline (%)	90.97	90.94	88.92	88.72	89.90	89.73
	Post-intervention (%)	93.02	92.28	91.42	89.52	92.17	90.89
	Change (%)	2.05	1.34	2.50	0.80	2.27	1.16
	OR (95% CI)	1.13 (0.99-1.29)		**1.27(1.09-1.48)**		**1.19 (1.08-1.30)**	
	p-values	0.063		**0.002**		**<0.001**	
It is healthier to drink plain boiled water than sugar-sweetened beverages. [yes]	Baseline (%)	85.35	85.67	83.56	83.66	84.64	84.96
	Post-intervention (%)	83.25	81.93	80.57	80.36	82.24	81.40
	Change (%)	-2.10	-3.74	-2.99	-3.30	-2.40	-3.66
	OR (95% CI)	**1.14 (1.04-1.25)**		1.02 (0.91-1.15)		**1.10 (1.03-1.18)**	
	p-values	**0.006**		0.685		**0.006**	
The food at the bottom of the dietary pagoda should be eaten more. [yes]	Baseline (%)	36.35	33.15	30.08	34.91	33.83	32.79
	Post-intervention (%)	39.31	39.06	24.30	24.55	44.21	40.18
	Change (%)	49.34	47.06	34.91	-10.36	10.38	7.39
	OR (95% CI)	**1.13 (1.04-1.23)**		**1.29 (1.15-1.45)**		**1.20 (1.12-1.27)**	
	p-values	**0.004**		**<0.001**		**<0.001**	
Obesity is bad for health. [yes]	Baseline (%)	83.13	81.83	77.16	73.88	80.59	77.88
	Post-intervention (%)	86.91	81.44	82.21	75.51	84.79	78.22
	Change (%)	3.78	-0.39	5.05	1.63	4.20	0.34
	OR (95% CI)	**1.56 (1.40-1.73)**		**1.35 (1.20-1.53)**		**1.45 (1.35-1.56)**	
	p-values	**<0.001**		**<0.001**		**<0.001**	
Belief							
Believe you can achieve an ideal weight status through effort. [true]	Baseline (%)	69.64	69.88	64.84	65.73	67.47	67.44
	Post-intervention (%)	72.89	70.56	69.32	67.03	71.18	68.61
	Change (%)	3.25	0.68	4.48	1.30	3.71	1.17
	OR (95% CI)	**1.17 (1.08-1.27)**		**1.20(1.08-1.33)**		**1.17 (1.10-1.24)**	

	p-values	**<0.001**		**0.001**		**<0.001**	
Eat more fruits and vegetables and less high-energy snacks to lose weight. [true]	Baseline (%)	45.97	44.02	47.41	47.50	46.48	45.13
	Post-intervention (%)	51.97	48.95	54.87	51.87	52.94	49.86
	Change (%)	6.00	4.93	7.46	4.37	6.46	4.73
	OR (95% CI)	1.07 (0.98-1.16)		**1.19(1.07-1.32)**		**1.11 (1.04-1.18)**	
	p-values	0.122		**0.001**		**0.001**	
Exercise to lose weight. [true]	Baseline (%)	49.36	48.67	48.99	50.67	49.34	49.26
	Post-intervention (%)	56.08	54.17	57.93	55.45	56.60	54.43
	Change (%)	6.72	5.50	8.94	4.78	7.26	5.17
	OR (95% CI)	1.07 (0.99-1.17)		**1.27 (1.14-1.40)**		**1.13 (1.06-1.20)**	
	p-values	0.079		**<0.001**		**<0.001**	
Practice							
Fruit [daily intake≥3 servings/day]	Baseline (%)	8.76	7.97	8.01	7.05	8.50	7.65
	Post-intervention (%)	10.51	8.70	9.53	7.59	10.05	8.19
	Change (%)	1.75	0.73	1.52	0.54	1.55	0.54
	OR (95% CI)	**1.14 (1.00-1.30)**		1.14 (0.96-1.35)		**1.14 (1.04-1.26)**	
	p-values	**0.043**		0.141		**0.007**	
Sugar-sweetened beverages [daily intake=0 cups/week]	Baseline (%)	34.38	34.09	31.14	31.60	32.81	32.80
	Post-intervention (%)	37.38	38.57	38.54	34.24	37.09	36.37
	Change (%)	3.00	4.48	7.40	2.64	4.28	3.57
	OR (95% CI)	0.91 (0.84-0.99)		**1.32 (1.19-1.47)**		1.04 (0.98-1.11)	
	p-values	0.031		**<0.001**		0.176	
Breakfast [frequency=7 days/week]	Baseline (%)	88.08	88.48	80.81	79.10	84.13	83.48
	Post-intervention (%)	89.12	88.29	82.85	79.20	85.69	83.74
	Change (%)	1.04	-0.19	2.04	0.10	1.56	0.26
	OR (95% CI)	**1.21 (1.06-1.37)**		**1.20 (1.06-1.37)**		**1.17 (1.08-1.27)**	
	p-values	**0.004**		**0.005**		**<0.001**	
Milk [frequency=7 days/week]	Baseline (%)	47.36	48.96	35.51	36.59	42.57	43.05
	Post-intervention (%)	50.58	48.39	39.51	36.49	46.19	42.81
	Change (%)	3.22	-0.57	4.00	-0.10	3.62	-0.24

	OR (95% CI)	**1.27 (1.17-1.37)**	**1.31 (1.17-1.45)**	**1.27 (1.20-1.35)**
	p-values	**< 0.001**	**< 0.001**	**< 0.001**

Note: Model was adjusted for age, sex, province, and urban/rural area. High school or above: Either the father or the mother is high school or above. Below high school: Both father and mother have below high school education

Table S13. Effectiveness on the student's knowledge, belief, and practice (by monthly household income).

Indicators	Time	≥2000		< 2000		Total	
		Intervention	Control	Intervention	Control	Intervention	Control
Knowledge							
It is necessary to exercise every day. [yes]	Baseline (%)	90.61	90.42	89.62	88.96	89.90	89.73
	Post-intervention (%)	92.96	91.66	91.05	90.65	92.17	90.89
	Change (%)	2.35	1.24	1.43	1.69	2.27	1.16
	OR (95% CI)	**1.21 (1.04-1.41)**		0.97 (0.71-1.34)		**1.19 (1.08-1.30)**	
	p-values	**0.016**		0.869		**<0.001**	
It is healthier to drink plain boiled water than sugar-sweetened beverages. [yes]	Baseline (%)	85.37	85.05	81.05	83.72	84.64	84.96
	Post-intervention (%)	82.32	81.17	80.46	81.65	82.26	81.30
	Change (%)	-3.05	-3.88	-0.59	-2.07	-2.40	-3.66
	OR (95% CI)	1.06 (0.95-1.19)		1.13 (0.89-1.43)		**1.10 (1.03-1.18)**	
	p-values	0.314		0.318		**0.006**	
The food at the bottom of the dietary pagoda should be eaten more. [yes]	Baseline (%)	32.36	31.61	25.78	26.47	33.83	32.79
	Post-intervention (%)	42.79	39.24	35.41	31.33	44.21	40.18
	Change (%)	10.43	7.63	9.63	4.86	10.38	7.39
	OR (95% CI)	**1.18 (1.07-1.31)**		**1.34 (1.08-1.68)**		**1.20 (1.12-1.27)**	
	p-values	**0.001**		**0.009**		**<0.001**	
Obesity is bad for health. [yes]	Baseline (%)	81.01	78.96	77.44	74.79	80.59	77.88
	Post-intervention (%)	85.53	79.07	82.47	76.11	84.79	78.22
	Change (%)	4.52	0.11	5.03	1.32	4.20	0.34
	OR (95% CI)	**1.54 (1.37-1.75)**		**1.39 (1.08-1.79)**		**1.45 (1.35-1.56)**	
	p-values	**<0.001**		**0.009**		**<0.001**	
Belief							
Believe you can achieve an ideal weight status	Baseline (%)	68.38	68.93	66.80	66.70	67.47	67.44
	Post-intervention (%)	72.87	69.63	71.51	67.80	71.18	68.61

through effort. [true]	Change (%)	4.49	0.70	4.71	1.10	3.71	1.17
	OR (95% CI)	**1.26(1.14-1.39)**		**1.25(1.01-1.55)**		**1.17 (1.10-1.24)**	
	p-values	**<0.001**		**0.041**		**<0.001**	
Eat more fruits and vegetables and less high-energy snacks to lose weight. [true]	Baseline (%)	47.42	46.52	50.46	47.99	46.48	45.13
	Post-intervention (%)	54.25	51.50	57.34	53.07	52.94	49.86
	Change (%)	6.83	4.98	6.88	5.08	6.46	4.73
	OR (95% CI)	**1.12 (1.01-1.23)**		1.11 (0.90-1.37)		**1.11 (1.04-1.18)**	
	p-values	**0.028**		0.325		**0.001**	
Exercise to lose weight. [true]	Baseline (%)	49.24	49.93	52.59	52.62	49.34	49.26
	Post-intervention (%)	56.69	55.18	60.07	57.22	56.60	54.43
	Change (%)	7.45	5.25	7.48	4.60	7.26	5.17
	OR (95% CI)	**1.14 (1.03-1.25)**		1.18 (0.96-1.46)		**1.13 (1.06-1.20)**	
	p-values	**0.010**		0.122		**<0.001**	
Practice							
Fruit [daily intake≥3 servings/day]	Baseline (%)	8.62	7.59	9.27	7.39	8.50	7.65
	Post-intervention (%)	10.47	8.59	11.09	7.39	10.05	8.19
	Change (%)	1.85	1.00	1.82	0.00	1.55	0.54
	OR (95% CI)	1.11 (0.95-1.30)		1.27 (0.91-1.77)		**1.14 (1.04-1.26)**	
	p-values	0.198		0.165		**0.007**	
Sugar-sweetened beverages [daily intake=0 cups/week]	Baseline (%)	31.82	32.38	36.70	33.17	32.81	32.80
	Post-intervention (%)	37.42	36.05	42.76	36.13	37.09	36.37
	Change (%)	5.60	3.67	6.06	2.96	4.28	3.57
	OR (95% CI)	**1.12 (1.02-1.24)**		1.19 (0.96-1.47)		1.04 (0.98-1.11)	
	p-values	**0.020**		0.104		0.176	
Breakfast [frequency=7 days/week]	Baseline (%)	83.65	83.76	80.97	79.28	84.13	83.48
	Post-intervention (%)	86.15	84.05	83.39	77.87	85.69	83.74
	Change (%)	2.50	0.29	2.42	-1.41	1.56	0.26
	OR (95% CI)	**1.31 (1.14-1.50)**		**1.43 (1.10-1.85)**		**1.17 (1.08-1.27)**	
	p-values	**<0.001**		**0.008**		**<0.001**	
Milk [frequency=7 days/week]	Baseline (%)	39.94	41.52	37.04	34.69	42.57	43.05
	Post-intervention (%)	44.22	41.69	43.13	34.79	46.19	42.81
	Change (%)	4.28	0.17	6.09	0.10	3.62	-0.24
	OR (95% CI)	**1.30 (1.17-1.43)**		**1.45 (1.17-1.80)**		**1.27 (1.20-1.35)**	
	p-values	**< 0.001**		**0.001**		**< 0.001**	

Note: Model was adjusted for age, sex, province, and urban/rural area. Monthly household income (RMB)

Table S14. Effectiveness on the parents' knowledge, belief, and practice (by Only child / Non-only child status).

Indicators	Time	Only child		Non-only child		Total	
		Intervention	Control	Intervention	Control	Intervention	Control
Knowledge							
It is necessary to exercise every day. [yes]	Baseline (%)	90.88	90.21	88.58	87.67	90.12	89.33
	Post-intervention (%)	91.35	91.06	89.60	89.27	90.77	90.43
	Change (%)	0.47	0.85	1.02	1.60	0.65	1.10
	OR (95% CI)	0.95 (0.84-1.07)		1.02 (0.88-1.17)		0.95 (0.86-1.04)	
	p-values	0.407		0.806		0.243	
It is healthier to drink plain boiled water than sugar-sweetened beverages. [yes]	Baseline (%)	95.19	95.04	93.50	93.23	94.63	92.42
	Post-intervention (%)	92.80	92.00	90.53	91.39	92.05	91.79
	Change (%)	-2.39	-3.04	-2.97	-1.84	-2.58	-2.63
	OR (95% CI)	1.09 (0.95-1.26)		0.85 (0.72-1.01)		1.00 (0.89-1.11)	
	p-values	0.198		0.071		0.930	
The food at the bottom of the dietary pagoda should be eaten more. [yes]	Baseline (%)	37.14	35.99	28.27	26.29	34.22	32.66
	Post-intervention (%)	43.13	42.55	34.76	32.61	40.37	39.14
	Change (%)	5.99	6.56	6.49	6.32	6.15	6.48
	OR (95% CI)	0.96 (0.89-1.04)		1.00 (0.89-1.12)		0.98 (0.91-1.04)	
	p-values	0.356		0.966		0.440	
Obesity is bad for health. [yes]	Baseline (%)	89.84	88.33	83.94	83.74	87.91	86.77
	Post-intervention (%)	89.39	88.48	84.94	83.51	87.93	86.79
	Change (%)	-0.45	0.15	1.00	-0.23	0.02	0.02
	OR (95% CI)	0.93 (0.83-1.04)		1.13 (0.98-1.30)		1.00 (0.92-1.09)	
	p-values	0.180		0.088		0.985	
Belief							
Don't buy drinks for children. [true]	Baseline (%)	44.75	45.58	42.61	44.59	44.04	45.24
	Post-intervention (%)	48.27	46.83	46.17	46.51	47.57	46.72
	Change (%)	3.52	1.25	3.56	1.92	3.53	1.48
	OR (95% CI)	**1.14 (1.05-1.23)**		**1.09 (0.99-1.21)**		**1.12 (1.06-1.19)**	
	p-values	**0.001**		**0.087**		**<0.001**	
Children take part in physical	Baseline (%)	74.67	76.89	76.12	77.30	75.15	77.03
	Post-intervention (%)	78.87	78.60	79.10	78.98	78.95	78.73

exercise with their classmates. [true]	Change (%)	4.20	1.71	2.98	1.68	3.80	1.70
	OR (95% CI)	**1.19 (1.10-1.30)**		1.09 (0.97-1.23)		**1.16 (1.08-1.24)**	
	p-values	**<0.001**		0.136		**<0.001**	
Set aside time every day for children to exercise. [true]	Baseline (%)	79.31	81.45	82.49	83.94	80.36	82.31
	Post-intervention (%)	83.23	83.89	84.84	85.11	83.77	84.31
	Change (%)	3.92	2.44	2.35	1.17	3.41	2.00
	OR (95% CI)	**1.12 (1.02-1.23)**		1.11 (0.98-1.26)		**1.12 (1.04-1.21)**	
	p-values	**0.017**		0.141		**0.004**	
Look at the nutrition label when shopping in the supermarket. [true]	Baseline (%)	70.84	71.47	71.84	72.80	71.17	71.93
	Post-intervention (%)	74.80	73.72	74.95	75.13	74.85	74.20
	Change (%)	3.96	2.25	3.11	2.33	3.68	2.27
	OR (95% CI)	**1.12 (1.03-1.22)**		1.05 (0.94-1.18)		**1.10 (1.03-1.18)**	
	p-values	**0.006**		0.387		**0.006**	
Drinks are not always available at home. [true]	Baseline (%)	68.48	69.27	68.27	69.12	68.41	69.22
	Post-intervention (%)	70.12	69.71	69.37	70.19	69.87	69.88
	Change (%)	1.64	0.44	1.10	1.07	1.46	0.66
	OR (95% CI)	1.08 (1.00-1.18)		1.00 (0.90-1.12)		1.05(0.99-1.13)	
	p-values	0.055		0.984		0.118	
Practice							
Fruit [daily intake≥3 servings/day]	Baseline (%)	5.97	5.27	7.08	6.17	6.34	5.58
	Post-intervention (%)	6.77	6.27	7.33	7.23	6.96	6.60
	Change (%)	0.80	1.00	0.25	1.06	0.62	1.02
	OR (95% CI)	0.95 (0.82-1.09)		0.86 (0.71-1.04)		0.92 (0.82-1.03)	
	p-values	0.457		0.118		0.130	
Sugar-sweetened beverages [daily intake≤4 cups/week]	Baseline (%)	92.06	92.78	91.60	92.39	91.91	92.65
	Post-intervention (%)	92.97	92.50	91.66	92.57	92.54	92.53
	Change (%)	0.91	-0.28	0.06	0.18	0.63	-0.12
	OR (95% CI)	**1.22 (1.07-1.39)**		0.98 (0.82-1.18)		**1.13 (1.02-1.26)**	
	p-values	**0.003**		0.827		**0.025**	
Breakfast [frequency=7 days/week]	Baseline (%)	85.89	86.71	83.11	84.52	84.97	85.96
	Post-intervention (%)	88.54	87.32	85.59	85.34	87.57	86.64
	Change (%)	2.65	0.61	2.48	0.82	2.60	0.68
	OR (95% CI)	**1.26 (1.14-1.40)**		**1.18 (1.02-1.36)**		**1.23 (1.13-1.34)**	
	p-values	**<0.001**		**0.023**		**<0.001**	
	Baseline (%)	30.08	29.18	24.08	24.66	28.10	27.63

Milk [frequency=7 days/week]	Post-intervention (%)	34.01	31.38	27.02	26.35	31.71	29.66
	Change (%)	3.93	2.20	2.94	1.69	3.61	2.03
	OR (95% CI)	**1.11 (1.02-1.20)**		**1.09 (0.97-1.22)**		**1.10 (1.03-1.18)**	
	p-values	**0.011**		**0.151**		**0.003**	

Note: Model was adjusted for age, sex, province, and urban/rural area.

Table S15. Effectiveness on the parents' knowledge, attitude, and behavior (by parental education).

Indicators	Time	High school or above		Below high school		Total	
		Intervention	Control	Intervention	Control	Intervention	Control
Knowledge							
It is necessary to exercise every day. [yes]	Baseline (%)	91.72	91.36	87.36	86.76	90.12	89.33
	Post-intervention (%)	92.01	92.18	88.63	88.09	90.77	90.43
	Change (%)	0.29	0.82	1.27	1.33	0.65	1.10
	OR (95% CI)	0.92 (0.81-1.05)		1.00 (0.87-1.15)		0.95 (0.86-1.04)	
	p-values	0.219		0.995		0.243	
It is healthier to drink plain boiled water than sugar-sweetened beverages. [yes]	Baseline (%)	95.27	95.37	93.80	93.31	94.63	94.42
	Post-intervention (%)	93.17	92.63	90.09	90.67	92.05	91.79
	Change (%)	-2.10	-2.74	-3.71	-2.64	-2.58	-2.63
	OR (95% CI)	1.12 (0.97-1.30)		0.86 (0.72-1.01)		1.00 (0.89-1.11)	
	p-values	0.135		0.067		0.930	
The food at the bottom of the dietary pagoda should be eaten more. [yes]	Baseline (%)	39.83	39.43	23.63	22.73	34.22	32.66
	Post-intervention (%)	46.01	45.80	29.78	29.32	40.37	39.14
	Change (%)	6.18	6.37	6.15	6.59	6.15	6.48
	OR (95% CI)	0.99 (0.91-1.07)		0.97 (0.86-1.08)		0.98 (0.91-1.04)	
	p-values	0.776		0.542		0.440	
Obesity is bad for health. [yes]	Baseline (%)	90.91	90.00	82.91	82.38	87.91	86.77
	Post-intervention (%)	90.44	89.73	83.70	82.67	87.93	86.79
	Change (%)	-0.47	-0.27	0.79	0.29	0.02	0.02
	OR (95% CI)	0.97 (0.86-1.10)		1.05 (0.92-1.20)		1.00 (0.92-1.09)	
	p-values	0.629		0.488		0.985	
Belief							

Don't buy drinks for children. [true]	Baseline (%)	44.84	45.77	42.90	44.67	44.04	45.24
	Post-intervention (%)	48.39	47.22	46.20	46.09	47.57	46.72
	Change (%)	3.55	1.45	3.30	1.42	3.53	1.48
	OR (95% CI)	**1.13 (1.04-1.22)**		1.10 (1.00-1.22)		**1.12 (1.06-1.19)**	
	p-values	**0.002**		0.053		**<0.001**	
Children take part in physical exercise with their classmates. [true]	Baseline (%)	74.64	76.86	76.54	77.61	75.15	77.03
	Post-intervention (%)	78.26	78.75	80.42	78.96	78.95	78.73
	Change (%)	3.62	1.89	3.88	1.35	3.80	1.70
	OR (95% CI)	**1.13 (1.03-1.23)**		**1.20 (1.07-1.35)**		**1.16 (1.08-1.24)**	
	p-values	**0.008**		**0.002**		**<0.001**	
Set aside time every day for children to exercise. [true]	Baseline (%)	79.34	82.01	82.29	82.98	80.36	82.31
	Post-intervention (%)	83.00	84.08	85.23	84.72	83.77	84.31
	Change (%)	3.66	2.07	2.94	1.74	3.41	2.00
	OR (95% CI)	**1.13 (1.02-1.25)**		1.11 (0.97-1.26)		**1.12 (1.04-1.21)**	
	p-values	**0.015**		0.116		**0.004**	
Look at the nutrition label when shopping in the supermarket. [true]	Baseline (%)	71.81	71.43	69.57	72.84	71.17	71.93
	Post-intervention (%)	75.21	74.09	74.00	74.25	74.85	74.20
	Change (%)	3.40	2.66	4.43	1.41	3.68	2.27
	OR (95% CI)	1.06 (0.97-1.15)		**1.21 (1.08-1.35)**		**1.10 (1.03-1.18)**	
	p-values	0.226		**0.001**		**0.006**	
Drinks are not always available at home. [true]	Baseline (%)	67.78	68.52	69.88	70.16	68.41	69.22
	Post-intervention (%)	69.91	69.54	70.03	70.34	69.87	69.88
	Change (%)	2.13	1.02	0.15	0.18	1.46	0.66
	OR (95% CI)	1.08 (0.99-1.18)		1.00 (0.90-1.11)		1.05 (0.99-1.13)	
	p-values	0.084		0.970		0.118	
Practice							
Fruit [daily intake≥3 servings/day]	Baseline (%)	6.10	5.40	6.58	5.81	6.34	5.58
	Post-intervention (%)	6.45	6.06	7.78	7.25	6.96	6.60
	Change (%)	0.35	0.66	1.20	1.44	0.62	1.02
	OR (95% CI)	0.934 (0.80-1.08)		0.94 (0.78-1.13)		0.92 (0.82-1.03)	
	p-values	0.354		0.519		0.130	
Sugar-sweetened beverages [daily	Baseline (%)	92.33	93.26	91.47	91.97	91.91	92.65
	Post-intervention (%)	93.20	92.93	91.68	92.00	92.54	92.53
	Change (%)	0.87	-0.33	0.21	0.03	0.63	-0.12

intake≤4 cups/week]	OR (95% CI)	**1.24 (1.07-1.43)**		**1.03 (0.86-1.22)**		**1.13 (1.02-1.26)**	
	p-values	**0.003**		**0.777**		**0.025**	
Breakfast [frequency=7 days/week]	Baseline (%)	86.64	87.83	82.24	83.62	84.97	85.96
	Post-intervention (%)	89.23	88.56	84.69	84.30	87.57	86.64
	Change (%)	2.59	0.73	2.45	0.68	2.60	0.68
	OR (95% CI)	**1.25(1.11-1.40)**		**1.23 (1.13-1.34)**		**1.23 (1.13-1.34)**	
	p-values	**<0.001**		**<0.001**		**<0.001**	
Milk [frequency=7 days/week]	Baseline (%)	30.80	29.53	22.88	24.59	28.10	27.63
	Post-intervention (%)	34.95	31.55	25.41	26.67	31.71	29.66
	Change (%)	4.15	2.02	2.53	2.08	3.61	2.03
	OR (95% CI)	**1.14 (1.05-1.23)**		1.04 (0.93-1.16)		**1.10 (1.03-1.18)**	
	p-values	**0.003**		0.533		**0.003**	

Note: Model was adjusted for age, sex, province, and urban/rural area. High school or above: Either the father or the mother is high school or above. Below high school: Both father and mother have below high school education
